# The genetic dissection of fetal haemoglobin persistence in sickle cell disease in Nigeria

**DOI:** 10.1093/hmg/ddae014

**Published:** 2024-02-10

**Authors:** Oyesola O Ojewunmi, Titilope A Adeyemo, Ajoke I Oyetunji, Bassey Inyang, Afolashade Akinrindoye, Baraka S Mkumbe, Kate Gardner, Helen Rooks, John Brewin, Hamel Patel, Sang Hyuck Lee, Raymond Chung, Sara Rashkin, Guolian Kang, Reuben Chianumba, Raphael Sangeda, Liberata Mwita, Hezekiah Isa, Uche-Nnebe Agumadu, Rosemary Ekong, Jamilu A Faruk, Bello Y Jamoh, Niyi M Adebiyi, Ismail A Umar, Abdulaziz Hassan, Christopher Grace, Anuj Goel, Baba P D Inusa, Mario Falchi, Siana Nkya, Julie Makani, Hafsat R Ahmad, Obiageli Nnodu, John Strouboulis, Stephan Menzel

**Affiliations:** School of Cancer and Pharmaceutical Sciences, King’s College London, 123 Coldharbour Lane, London SE5 9NU, United Kingdom; Department of Non-Communicable Disease Epidemiology, London School of Hygiene and Tropical Medicine, Keppel Street, London WC1E 7HT, United Kingdom; Department of Haematology and Blood Transfusion, College of Medicine, University of Lagos, P.M.B 12003, Lagos, Nigeria; Sickle Cell Foundation Nigeria, Ishaga Road, Idi-Araba, P.O. Box 3463, Lagos, Nigeria; Department of Medical Biochemistry, College of Health Sciences, University of Abuja, Mohammed Maccido Road, Airport Road, P.M.B 117, Abuja, Nigeria; Sickle Cell Foundation Nigeria, Ishaga Road, Idi-Araba, P.O. Box 3463, Lagos, Nigeria; School of Science, University of Greenwich, Central Avenue, Chatham Maritime, Kent ME4 4TB, United Kingdom; Department of Biochemistry and Molecular Biology, Muhimbili University of Health and Allied Sciences, P.O. Box 65001, United Nations Rd, Dar es Salaam, Tanzania; Department of Artificial Intelligence and Innovative Medicine, Tohoku University Graduate School of Medicine, 980-8573, 2-1 Seiryo-machi, Aoba-ku, Sendai, Miyagi, Japan; School of Cancer and Pharmaceutical Sciences, King’s College London, 123 Coldharbour Lane, London SE5 9NU, United Kingdom; Clinical Haematology, Haematology and Oncology Directorate, Guy’s Hospital, Great Maze Pond, London SE1 9RT, United Kingdom; School of Cancer and Pharmaceutical Sciences, King’s College London, 123 Coldharbour Lane, London SE5 9NU, United Kingdom; School of Cancer and Pharmaceutical Sciences, King’s College London, 123 Coldharbour Lane, London SE5 9NU, United Kingdom; Department of Haematological Medicine, King's College Hospital, London SE5 9RS, United Kingdom; NIHR BioResource Centre Maudsley, NIHR Maudsley Biomedical Research Centre (BRC) at South London and Maudsley NHS Foundation Trust (SLaM) and Institute of Psychiatry, Psychology and Neuroscience (IoPPN), King’s College London, 16 De Crespigny Park, London SE5 8AB, United Kingdom; NIHR BioResource Centre Maudsley, NIHR Maudsley Biomedical Research Centre (BRC) at South London and Maudsley NHS Foundation Trust (SLaM) and Institute of Psychiatry, Psychology and Neuroscience (IoPPN), King’s College London, 16 De Crespigny Park, London SE5 8AB, United Kingdom; NIHR BioResource Centre Maudsley, NIHR Maudsley Biomedical Research Centre (BRC) at South London and Maudsley NHS Foundation Trust (SLaM) and Institute of Psychiatry, Psychology and Neuroscience (IoPPN), King’s College London, 16 De Crespigny Park, London SE5 8AB, United Kingdom; St. Jude Children's Research Hospital, 262 Danny Thomas Place, Memphis, Tennessee 38105, United States; St. Jude Children's Research Hospital, 262 Danny Thomas Place, Memphis, Tennessee 38105, United States; Centre of Excellence for Sickle Cell Disease Research and Training (CESRTA), University of Abuja, Mohammed Maccido Road, Airport Road, P.M.B 117, Abuja, Nigeria; Department of Pharmaceutical Microbiology, Muhimbili University of Health and Allied Sciences, P.O. Box 65001, Dar es Salaam, Tanzania; Department of Pharmaceutical Microbiology, Muhimbili University of Health and Allied Sciences, P.O. Box 65001, Dar es Salaam, Tanzania; Centre of Excellence for Sickle Cell Disease Research and Training (CESRTA), University of Abuja, Mohammed Maccido Road, Airport Road, P.M.B 117, Abuja, Nigeria; Department of Haematology and Blood Transfusion, University of Abuja Teaching Hospital, Gwagwalada, P.M.B. 228, Gwagwalada, FCT Abuja, Nigeria; Department of Paediatrics, College of Health Sciences, University of Abuja, Mohammed Maccido Road, Airport Road, P.M.B 117, Abuja, Nigeria; Research Department of Genetics, Evolution and Environment, University College London, Gower Street, London WC1E 6BT, United Kingdom; Department of Paediatrics, Ahmadu Bello University/Ahmadu Bello University Teaching Hospital, P.M.B 006, Zaria, Nigeria; Department of Internal Medicine, Ahmadu Bello University/Ahmadu Bello University Teaching Hospital, P.M.B 006, Zaria, Nigeria; Department of Paediatrics, Ahmadu Bello University/Ahmadu Bello University Teaching Hospital, P.M.B 006, Zaria, Nigeria; Department of Biochemistry, Faculty of Life Sciences, Ahmadu Bello University, Sokoto Road, Samaru, P.M.B 006, Zaria, Nigeria; Department of Haematology and Blood Transfusion, Ahmadu Bello University, Sokoto Road, Samaru, P.M.B 006, Zaria, Nigeria; Division of Cardiovascular Medicine, Radcliffe Department of Medicine, University of Oxford, Centre for Human Genetics, Roosevelt Drive, Oxford OX37BN, United Kingdom; Division of Cardiovascular Medicine, Radcliffe Department of Medicine, University of Oxford, Centre for Human Genetics, Roosevelt Drive, Oxford OX37BN, United Kingdom; Evelina London Children’s Hospital, Guy’s and St. Thomas’ NHS Foundation Trust, Westminster Bridge Rd, London SE1 7EH, United Kingdom; Department of Twin Research and Genetic Epidemiology, King’s College London, St Thomas’ Hospital, Westminster Bridge Road, London SE1 7EH, United Kingdom; Department of Biochemistry and Molecular Biology, Muhimbili University of Health and Allied Sciences, P.O. Box 65001, United Nations Rd, Dar es Salaam, Tanzania; Tanzania Human Genetics Organisation, Sickle Cell Centre, 1 Kipalapala Street, Dar es Salaam, Tanzania; Sickle Cell Program, Muhimbili University of Health and Allied Sciences, P.O. Box 65001, United Nations Rd, Dar es Salaam, Tanzania; Department of Haematology and Blood Transfusion, Muhimbili University of Health and Allied Science, P.O. Box 65001, Dar es Salaam, Tanzania; Sickle Cell Program, Muhimbili University of Health and Allied Sciences, P.O. Box 65001, United Nations Rd, Dar es Salaam, Tanzania; Department of Haematology and Blood Transfusion, Muhimbili University of Health and Allied Science, P.O. Box 65001, Dar es Salaam, Tanzania; Centre for Haematology, Department of Immunology & Inflammation, Imperial College London, Commonwealth Building, Hammersmith Campus, Du Cane Rd, London W12 0NN, United Kingdom; Department of Paediatrics, Ahmadu Bello University/Ahmadu Bello University Teaching Hospital, P.M.B 006, Zaria, Nigeria; Centre of Excellence for Sickle Cell Disease Research and Training (CESRTA), University of Abuja, Mohammed Maccido Road, Airport Road, P.M.B 117, Abuja, Nigeria; Department of Haematology and Blood Transfusion, University of Abuja Teaching Hospital, Gwagwalada, P.M.B. 228, Gwagwalada, FCT Abuja, Nigeria; School of Cancer and Pharmaceutical Sciences, King’s College London, 123 Coldharbour Lane, London SE5 9NU, United Kingdom; School of Cancer and Pharmaceutical Sciences, King’s College London, 123 Coldharbour Lane, London SE5 9NU, United Kingdom

**Keywords:** fetal haemoglobin, sickle cell disease, genome-wide association study, heritability, haplotype analysis

## Abstract

The clinical severity of sickle cell disease (SCD) is strongly influenced by the level of fetal haemoglobin (HbF) persistent in each patient. Three major HbF loci (*BCL11A*, *HBS1L-MYB*, and *Xmn1-HBG2*) have been reported, but a considerable hidden heritability remains. We conducted a genome-wide association study for HbF levels in 1006 Nigerian patients with SCD (HbSS/HbSβ^0^), followed by a replication and meta-analysis exercise in four independent SCD cohorts (3,582 patients). To dissect association signals at the major loci, we performed stepwise conditional and haplotype association analyses and included public functional annotation datasets. Association signals were detected for *BCL11A* (lead SNP *rs6706648*, β = −0.39, *P* = 4.96 × 10^−34^) and *HBS1L-MYB* (lead SNP *rs61028892*, β = 0.73, *P* = 1.18 × 10^−9^), whereas the variant allele for *Xmn1-HBG2* was found to be very rare. In addition, we detected three putative new trait-associated regions. Genetically, dissecting the two major loci *BCL11A* and *HBS1L-MYB*, we defined trait-increasing haplotypes (*P* < 0.0001) containing so far unidentified causal variants. At *BCL11A*, in addition to a haplotype harbouring the putative functional variant rs1427407-‘T’, we identified a second haplotype, tagged by the rs7565301-‘A’ allele, where a yet-to-be-discovered causal DNA variant may reside. Similarly, at *HBS1L-MYB*, one HbF-increasing haplotype contains the likely functional small indel rs66650371, and a second tagged by rs61028892-‘C*’* is likely to harbour a presently unknown functional allele. Together, variants at *BCL11A* and *HBS1L-MYB* SNPs explained 24.1% of the trait variance. Our findings provide a path for further investigation of the causes of variable fetal haemoglobin persistence in sickle cell disease.

## Graphical abstract

**Figure:**
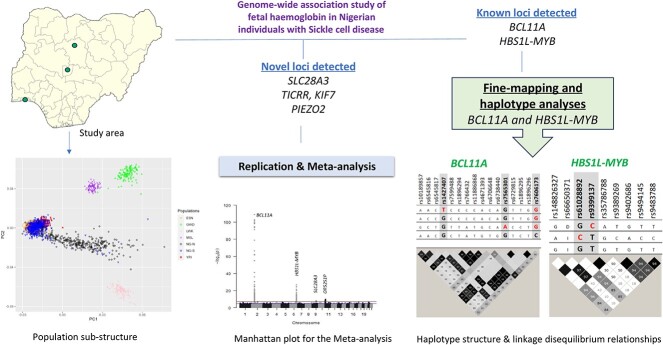

## Introduction

Sickle cell disease (SCD) is an inherited blood disorder that affects approximately 300 000 newborns globally every year. Half of these are born in Nigeria alone, where SCD is a major public health problem and contributes significantly to childhood morbidity and mortality, as fifty percent of children do not live beyond their 5^th^ birthday [1].

Polymerization of haemoglobin S (HbS) under hypoxic conditions is central to the pathophysiologic events in SCD, giving rise to many debilitating complications, including vaso-occlusive crises, stroke, and leg ulcers.

A wide variability in severity and clinical presentation is driven partially by additional genetic factors, such as the genotypic state at the site of mutation (homozygous: Hb SS; compound heterozygous: Hb SC, HbS beta thalassaemia) and by genetic disease modifiers at the beta-globin gene locus or elsewhere in the genome. To discover the underlying genes and genetic variants is highly desirable; however, the immense complexity of clinical presentations and pathological pathways involved has been a significant obstacle. A major breakthrough has been the discovery that a substantial persistence of fetal haemoglobin (HbF, α_2_γ_2_) in certain patients can alleviate the disease severity and improve survival [2]. This led to intense efforts to find ways to induce HbF expression therapeutically.

To a variable extent, some HbF persists naturally in adults, forming a quantitative trait with up to 89% heritability in the general population and 60%–70% in SCD patients [3, 4]. Since it has been challenging to assemble sufficiently large and homogeneous SCD study cohorts, the first HbF loci, and thus the first SCD modifier genes, outside the beta-globin gene cluster, HBS1 like Translational GTPase-*MYB* Proto-Oncogene (*HBS1L-MYB)* and B-cell lymphoma/leukaemia 11A (*BCL11A)* were discovered through studies in an extended family [5] and large non-anaemic population samples [6–8]. They were subsequently replicated in SCD patient cohorts [9]. Together, the three major HbF loci, the β-globin gene cluster on chromosome 11 (*Xmn1-HBG2*—a gamma-globin promoter polymorphism), *BCL11A* on chromosome 2, and *HBS1L-MYB* on chromosome 6, account for about 50% of HbF variability in healthy persons and 20%–30% in individuals with SCD in many populations [7, 9–13]. Therefore, half of the overall trait heritability is still unexplained, probably due to a lack of statistical power to detect large numbers of rare or weak-effect variants. To overcome this, to discover novel HbF or SCD severity loci and to better dissect the known ones, statistical power can be enhanced by assembling larger patient cohorts, by combining existing studies, or by ‘genetic loading’, i.e. recruiting subjects with extreme trait expression or marked family history. With its many patients, Nigeria provides unique opportunities in this respect. The enhanced haplotype resolution seen in African populations is expected to support fine-mapping, and the exceptional ethnic diversity of Nigeria will allow the evaluation of genetic variants that are rare elsewhere. Here, we present a first genome-wide study of fetal haemoglobin in patients with SCD in Nigeria, searching for new loci and performing fine-mapping to identify candidate functional variants and gain further insight into HbF regulation and heritability in persons with SCD.

## Results

### Genome-wide association analysis in the Nigerian cohort

We conducted a genome-wide association analysis for 475 male and 531 female patients with sickle cell disease (HbSS/HbSβ^0^) recruited in Lagos (South-West Nigeria), Abuja (North-Central Nigeria), and Zaria (North-West Nigeria). The median HbF of the patients was 6.69% (range: 0.8–32%) (Supplementary Table 1), and the distribution of HbF is shown in Supplementary Fig. 1. The population structure shows the diversity of the patients recruited for this study (Supplementary Fig. 2).

**Figure 1:**
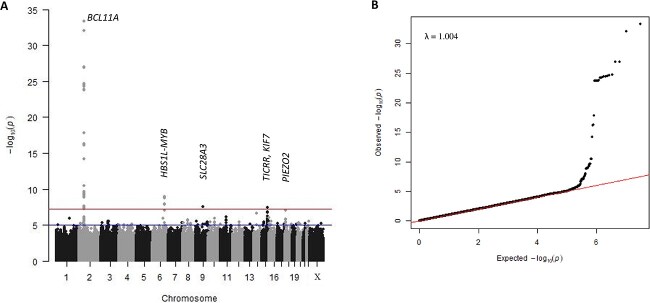
(A) Manhattan plot of -log_10_(P-values). The upper line indicates the threshold for genome-wide significance (*P* < 5 × 10^−8^), the lower line for suggestive association (*P* < 1 × 10^−6^). (B) Quantile-quantile plot of -log10(P-values) for all SNPs tested. Population structure is implied to be effectively controlled since there is no deviation from the null hypothesis line except at the tail end. The λ_GC_ value (lambda genomic control inflation factor) of 1.004 indicates that population stratification has been adjusted effectively by the model and relationship matrix. Alt-text. (A). This shows strong genetic association signals for *BCL11A* (*P* = 4.96 × 10^−34^) and *HBS1L-MYB* (*P* = 1.18 × 10^−9^) on chromosomes 2 and 6 respectively. In addition, we identified three novel loci, *SLC28A3* on chromosome 9 (*P* = 2.52 × 10^−8^), *TICRR/KIF7* on chromosome 15 (*P* = 3.34 × 10^−8^), and *PIEZO2* on chromosome 18 (*P* = 8.04 × 10^−8^). (B). The quantile-quantile plot aligns with the null hypothesis line, deviating only at the tail end, representing the associated loci.

We detected two of the three major HbF modifier loci (Fig. 1, Table 1), *BCL11A* (lead SNP *rs6706648*, β = −0.39, *P* = 4.96 × 10^−34^) and *HBS1L-MYB* (lead SNP *rs61028892*, β = 0.73, *P* = 1.18 × 10^−9^). The third, *XmnI-HBG2* (rs7482144), was not included in the association analysis with our trait measure since this critical variant at the beta-globin gene cluster was rare (MAF = 0.007), consistent with a previous finding [14]. A list of genome-wide significant SNPs reported to be associated with HbF in previous studies and this current study is shown in Supplementary Table 3.

**Table 1:** List of genome-wide loci in Nigeria cohort and the replication cohorts.

					Nigeria cohort = 1006			Replication meta-analysis			Meta-analysis of Nigerian and replication cohorts		
Chr	Locus	SNP	Position	EA/OA	EAF	Β	p-value	EAF	N	p-value	EAF	N	p-value
2	*BCL11A*	rs6706648	60 494 905	T/C	0.41	−0.39	4.96 × 10^−34^	0.42	2533	2.47 × 10^−60^	0.42	3539	7.08 × 10^−92^
6	*HBS1L-MYB*	rs61028892	135 097 526	C/G	0.02	0.73	1.18 × 10^−9^	0.02	2509	4.92 × 10^−17^	0.02	3510	5.03 × 10^−25^
9	*SLC28A3*	rs115555854^*^	84 339 933	A/C	0.01	−0.73	2.52 × 10^−8^				0.01	1003	2.73 × 10^−8^
15	*TICRR, KIF7*	rs140496989	89 619 296	A/G	0.05	−0.43	3.34 × 10^−8^	0.04	2562	0.2216	0.04	3566	0.06
18	*PIEZO2*	rs58817161	10 818 373	C/T	0.02	−0.63	8.04 × 10^−8^	0.02	2511	0.7841	0.02	3517	0.00197

Chr: Chromosome; SNP: reference ID for the Single Nucleotide Polymorphism; EA: Effect allele; OA: Other allele; EAF: Effect allele frequency; **β**: allelic effect size; N = sample size. The replication meta-analysis includes SCD patients’ datasets from the UK, Tanzania, and African-American cohorts. Where the allelic effect size (β) is positive, the HbF-boosting allele is the alternative allele; where it is negative, HbF is increased by the reference allele. Chromosomal positions are in hg38. *MAF for rs115555854 was less than 1% in the UK, Tanzania, and African-American cohorts.

In addition, we detected two novel association signals at a genome-wide significance level: *SLC28A3* on chromosome 9 (*rs115555854*: β = −0.73, *P* = 2.52 × 10^−8^) and *TICRR/KIF7* on chromosome 15 (*rs140496989*: β = −0.43, *P* = 3.34 × 10^−8^). A third locus, *PIEZO2* on chromosome 18, showed association at a 5% false discovery rate (p-value < 1.37 × 10^−7^) (*rs58817161*: β = −0.63, *P* = 8.04 × 10^−8^) (Table 1). Our functional annotations for these novel loci using fGCTA (Supplementary Figs 3–5) did not reveal a possible functional SNP.

### Genetic dissection of the *BCL11A* and *HBS1L-MYB* loci

**Figure 2:**
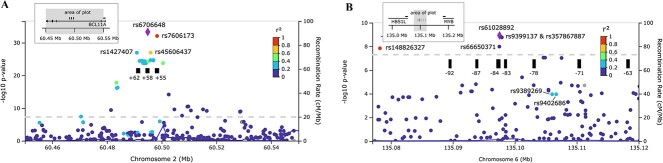
Regional association plots for *BCL11A* (A) and *HBS1L-MYB* loci (B). The dashed line shows the threshold for genome-wide significance (p < 5 × 10^−8^). The black rectangular blocks show key erythroid regulatory elements, detected as DNase I hypersensitive sites for *BCL11A* [29] and LDB1 binding sites for *HBS1L-MYB* [33]. Alt-text. (A). The genome-wide significant *BCL11A* SNPs overlap with the erythroid enhancer elements and DNase I hypersensitive sites (DHSs), denoted as +55 kb, +58 kb, and + 62 kb sites relative to the transcription start sites with their proxy SNPs: rs7606173, rs6706648, and rs1427407 respectively. (B). Similar to the *BCL11A* SNPs, the *HBS1L-MYB* intergenic region contains proxy SNPs that fall within the erythroid regulatory elements with rs61028892 and rs66650371 tagging +84 kb DHS.

Both loci overlap erythroid enhancer elements for adjacent genes *BCL11A* and *MYB* (Fig. 2). They contain multiple independent association signals, indicating the likely presence of several functionally active DNA variants interfering with enhancer function. Our fine-mapping strategy aimed to define the haplotypes carrying HbF-boosting alleles and identify putative functional variants.

First, we chose variants tagging HbF-relevant haplotypes aided by conditional association analysis. We then surveyed our phase-aligned genotype data to identify regional haplotypes defined by these variants, recorded their presence in patients, and measured their effects. In our fine-mapping, we included functional sequence annotation to spotlight putative functional variants using functional GCTA (fGCTA). To differentiate between variants in close linkage disequilibrium in African datasets that appeared to have a similar impact on HbF values, we performed additional association analysis in a European non-anaemic (TwinsUK) cohort.

***BCL11A*:** Stepwise regression analysis initially yielded two partially independent signals, rs6706648 (our lead SNP at *BCL11A*) and rs1427407 (Table 2), a variant widely reported in other cohorts [15] shown to disrupt critical transcription factor sites and are considered functional [16]. The fGCTA approach identified the same variants as in the standard conditional analysis (Table 2) for the erythroblast GATA1, K562, and HUDEP-2 annotations; rs1427407 overlapped with a GATA1 site (Supplementary Table 4, Supplementary Fig. 6). When we conditioned on rs1427407 alone (Supplementary Table 5), the most significant SNP obtained was not rs6706648 but rs7565301, which is less frequent but has a similar allelic effect. To define haplotypes of interest, we also considered rs7606173, a widely reported SNP [17] that we found in close LD with our lead SNP (D′ = 1, r^2^ = 0.91) (Fig. 2). Accordingly, the power to distinguish between these two SNPs in our patient dataset is limited. Somewhat surprisingly, the LD between them is significantly less tight in European populations (EUR: D′ = 0.99, r^2^ = 0.61 versus YRI: D′ = 1.0, r^2^ = 0.91 in 1,000 Genomes Phase III data implemented in LDlink [18]. Therefore, we decided to test both for HbF association (ln[%F-cells] trait) in the TwinsUK dataset and found rs7606173 to be substantially stronger (β = − 0.296, p = 2.29 × 10^−79^ vs. β = − 0.243, p = 1.27 × 10^−47^ of rs6706648) (Supplementary Table 6).

**Table 2:** Conditional analysis of chromosome 2 in the Nigerian cohort.

				Non-conditional		Conditional on rs6706648		Conditional on rs6706648 + rs1427407	
Chr	SNP	Position	EA/EAF	β-value	p-value	β-value	p-value	β-value	p-value
2	rs6706648	60 494 905	T/0.41	−0.395	4.96 × 10^−34^				
2	rs1427407	60 490 908	T/0.25	0.41	1.14 × 10^−27^	0.20	3.68 × 10^−7^		
2	rs58789059	60 509 717	A/0.17	−0.28	3.03 × 10^−11^	−0.12	0.005	−0.12	0.004

Chr: Chromosome; SNP: reference ID for the Single Nucleotide Polymorphism; EA: Effect allele; OA: Other allele; EAF: Effect allele frequency; **β**: allelic effect size. Chromosomal positions are in hg38.

**Figure 3:**
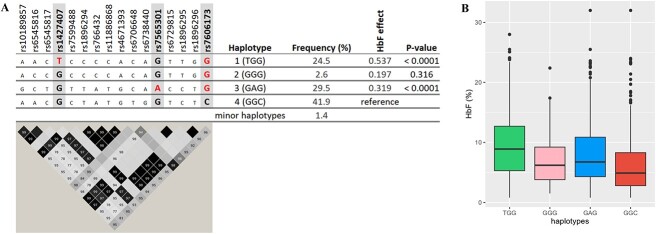
Major haplotypes at the *BCL11A* locus are defined by critical markers rs1427407, rs7565301, and rs7606173. A: shows the relationship of these tagging SNPs with other variants populating these haplotypes, i.e. associated alleles (top) and LD relationships (bottom); the effect alleles ‘T’, ‘A’, and ‘G’ for rs1427407, rs7565301, and rs7606173 are highlighted in red . LD plot: Each diamond shows the value of D′, and the LD indicates white (r^2^ = 0), shades of grey (0 < r^2^ < 1), and black (r^2^ = 1). B: shows haplotype effects on HbF levels. Alt-text. (A). Two haplotypes (haplotypes 1 and 3) with strong HbF-increasing effects, as defined by rs1427407, rs7565301, and rs7606173, were detected at the *BCL11A* locus. Haplotype 1: TGG carries HbF-boosting alleles at rs1427407 (‘T’) and rs7606173 (‘G’) while haplotype 3 carries rs7565301 variant (‘A’ allele) and rs7606173 (‘G’). These two haplotypes showed significant association with HbF compared with haplotype 4, carrying low-HbF variants (*P*-values < 0.0001). (B). *BCL11A* haplotypes 1 and 3 showed the highest levels of fetal haemoglobin.

We used SNPs rs1427407, rs7565301, and rs7606173 to define haplotypes at *BCL11A*, of which we observed six, four of them with an allele frequency above 1% (Fig. 3). We estimated the effect of the common *BCL11A* haplotypes on our trait (ln [HbF%]) compared to ‘basal’ haplotype 4, which contains low-HbF alleles for all three tag SNPs (‘GGC’). Haplotype 1 (‘**T**GG’), containing the high-HbF allele for rs1427407, had a significant trait-increasing effect (+0.537, *P* < 0.0001) as did haplotype 3 (‘G**A**G’), which contains the high-HbF allele for rs7565301 (+0.319, *P* < 0.0001). Haplotype 2 (‘GG**G**’), which contains the high-HbF allele only for rs7606173, had a smaller effect (+0.197), which was not significant, possibly due to its low frequency of 2.6%. Haplotypes 2 and 3 did not have significantly different effects. These HbF-boosting haplotype effects were validated in the UK SCD datasets (Supplementary Table 7). Candidate causative SNPs for each haplotype were identified. For haplotype 1, its tag SNP (rs1427407) itself is likely to be functional [16]. For haplotype 3, credible SNPs are the tag SNP rs7565301 and variants in close LD with it (rs6729815: D′ = 1.0, r^2^ = 1.0; rs7599488: D′ = 1.0, r^2^ = 1.0; rs6545817: D′ = 0.98, r^2^ = 0.96; rs10189857: D′ = 1.0, r^2^ = 0.98; rs6545816: D′ = 0.98, r^2^ = 0.73 (Fig. 3).

**Figure 4:**
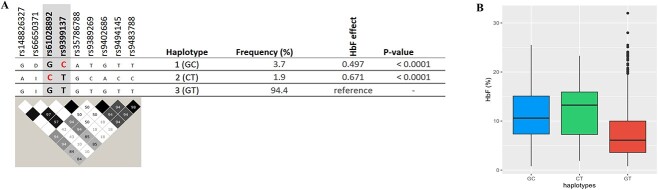
Major haplotypes at the HBS1L-MYB locus are defined by critical markers rs61028892 and rs9399137. A: shows the relationship of these tagging SNPs with other variants populating these haplotypes, i.e. associated alleles (top) and LD relationships (bottom). rs66650371: D and I represent 3-bp deletion and insertion, respectively. The effect allele ‘C’ for rs61028892 and rs9399137 is highlighted in red. LD plot: Each diamond shows the value of D′, and the LD indicates white (r^2^ = 0), shades of grey (0 < r^2^ < 1), and black (r^2^ = 1). B: shows haplotype effects on HbF levels. Alt-text (A). Two major HbF-boosting haplotypes were identified at the *HBS1L-MYB* locus as defined by rs9399137 and rs61028892: namely, haplotypes 1 (carrying the high-HbF C-allele at rs9399137) and 2 (carrying the high-HbF C-allele at rs61028892). (B). This demonstrates levels of fetal haemoglobin according to the haplotypes identified; haplotypes 1 and 2 show significantly (P < 0.0001) higher levels of HbF compared to the low-HbF-carrying haplotype 3 (GT).

***HBS1L-MYB:*** Conditional analysis detected two separate association signals, the lead SNP, rs61028892, and rs9399137 (Table 3). fGCTA also identified rs61028892 as the peak SNP. The K562 annotation identified the same secondary signal (rs9399137) with the conditional analysis. However, erythroblast GATA1 and HUDEP-2 tracks identified a different secondary signal (rs66650371) (Supplementary Table 8*,*  Supplementary Fig. 7). The ‘basal’ haplotype 3 (‘GT’) (Fig. 4), carrying low-HbF variants at both positions, was overwhelmingly frequent (94%), as it is typical for African populations [19]. We observed two other haplotypes: haplotype 1 (‘G**C**’, carrying the high-HbF allele at rs9399137) with an HbF increasing effect of +0.497 (*P* < 0.0001) and haplotype 2 (‘**C**T’, carrying the high-HbF allele at rs61028892) with an effect of +0.671 (*P* < 0.0001). Similar haplotype effects were confirmed in the UK SCD datasets, as shown in Supplementary Table 9. At haplotype 1, a 3-bp deletion (rs66650371) in close LD (D′ = 1.0, r^2^ = 1.0) with the tag SNP for haplotype 1 (rs9399137) has previously been suggested to be functional [20, 21]. For haplotype 2, candidates for being causative are the tag SNP rs61028892 and variants in close LD with it (rs148826327: D′ = 1.0, r^2^ = 1.0, rs116460276: r^2^ = D′ = 1.0, r^2^ = 1.0) or an undetected variant (Fig. 4).

**Table 3:** Conditional analysis of chromosome 6 in the Nigerian cohort.

				Non-conditional		Conditional on rs61028892		Conditional on rs61028892 + rs9399137	
Chr	SNP	Position	EA/EAF	β-value	p-value	β-value	p-value	β-value	p-value
**6**	rs61028892	135 097 526	C/0.02	0.73	1.18 × 10^−9^				
6	rs9399137	135 097 880	C/0.04	0.50	1.71 × 10^−9^	0.52	9.48 × 10^−10^		
6	rs1135205	134 960 657	C/0.25	0.12	0.00079	0.12	0.00087	0.14	0.00013

Chr: Chromosome; SNP: reference ID for the Single Nucleotide Polymorphism; EA: Effect allele; OA: Other allele; EAF: Effect allele frequency; **β**: allelic effect size. Chromosomal positions are in hg38.

### Trait variability explained and SNP heritability.

We applied a multiple linear regression modelling to estimate the genetic effects on HbF (ln [HbF%]) using five representative and independent *BCL11A* (rs7606173, rs1427407, rs6738440*)* and *HBS1L-MYB* (rs9399137 and rs61028892) SNPs. For *BCL11A*, we selected two independent SNPs included in our haplotype association analysis (rs7606173 and rs1427407), which are the major SNPs overlapping the +55 and + 62 DNase I hypersensitive sites (DHS) of *BCL11A* enhancer. In addition, we included a third SNP, rs6738440, located within the +58 *BCL11A* DHS. Similarly, we included two independent *HBS1L-MYB* SNPs detected in this study (rs61028892 and rs9399137) that fall within the DHS enhancer. Taken together, these SNPs account for 24.1% HbF variance in the Nigerian SCD cohort. We further estimated genome-wide SNP heritability of HbF in the Nigerian cohort and obtained 0.44 (standard error: 0.52).

### Testing for replication of novel association signals

A list of all genome-wide and suggestive loci detected in the Nigerian cohort is presented in Supplementary Table 10. The regional association plots for the novel genome-wide significant loci are shown in Supplementary Figs 8–10.

For any novel locus detected near (above or below) the acknowledged threshold for genome-wide significance, there is a chance of it being either a true (caused by functional DNA sequence changes) or a spurious association signal. To distinguish these scenarios, we sought to confirm (‘replicate’) our three novel signals in three further sickle cell populations of African descent, combined through meta-analysis (Table 1). We detected no significant association, but the longitudinal data of 326 SCD African-American patients from St Jude Children’s Research Hospital showed a replication for *PIEZO2* (*P* = 0.00028) (Supplementary Table 11).

**Figure 5:**
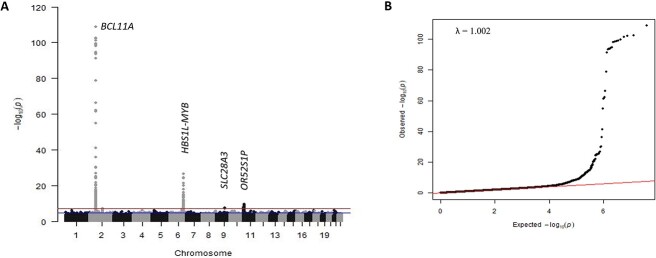
(A) Manhattan plot showing meta-analysis of 3582 SCD patients. The y-axis indicates the −log(*P*-value) for each variant in the meta-analysis, and the x-axis shows the chromosomal position. (B) Quantile-quantile plot of the observed vs expected *P*-values with the genomic control inflation factor (λ) of 1.002. Alt-text. (A). This shows genetic association signals for *BCL11A* on chromosome 2 (*P =* 1.83 × 10^−109^), *HBS1L-MYB* on chromosome 6 (*P =* 1.77 × 10^−27^), *SLC28A3* on chromosome 9 (*P* = 2.73 × 10^–8)^ and *OR52S1P* on chromosome 11 (*P =* 2.03 × 10^−10^). (B). This is a quantile-quantile plot of -log10 (P-values) for all SNPs tested, showing the distributions of the observed P-values from the genome-wide association study against the null hypothesis.

We conducted a further meta-analysis involving the genome-wide association results of all 3582 patients (Nigeria, UK, Tanzania, and African-American) to search for additional association signals. We identified one genome-wide significant locus and nine suggestive genome-wide significant loci in addition to those detected in the Nigerian cohort alone (Fig. 5, Supplementary Table 12).

## Discussion

We conducted a genome-wide quantitative-trait association study for fetal-haemoglobin levels in 1,006 Nigerian patients with sickle cell disease recruited from three regions of the country. At the genome-wide significance level, we detected two of the three known major loci for HbF persistence [7, 9, 22], *BCL11A* (chromosome 2) and *HBS1L-MYB* (*HMIP*) (chromosome 6), and three new loci, *SLC28A3* on chromosome 9, *TICRR/KIF7* on chromosome 15, and *PIEZO2* on chromosome 18. This is the first genome-wide association study for this trait in a West African population. Building upon a previous, smaller study that focused on the three known major HbF modifier loci (*BCL11A, HBS1L-MYB*, and *Xmni-HBG2*), we used the increased sample size, the genome-wide approach with the African-optimized dense marker set and, crucially, the ability to adjust for population structure, to dissect the genetic architecture of the fetal haemoglobin trait in the Nigerian patient population.

HbF persistence is a widely studied genetic trait in sickle cell patients due to its important disease-ameliorating and life-prolonging effect, the broad availability of a reliable assay system (high-performance liquid chromatography - HPLC), and its reduced complexity compared to other SCD-severity-related traits. HbF reactivation is a key therapeutic goal, targeted not only by the disease-modifying drug hydroxyurea but also by various gene therapy approaches recently approved for clinical use or undergoing clinical trials [23, 24]. *BCL11A* and *HBS1L-MYB* are major HbF loci present in all human populations, including in SCD patients [7, 9, 22], but the third, *Xmn1-HBG2*, residing within the beta-globin gene cluster, is detected only in patients where so-called ‘Senegal’ and ‘Arab-Indian’ haplotypes surrounding the sickle mutation are prevalent. While it was unsurprising to encounter *BCL11A* and *HBS1L-MYB* in the current study, the genetic dissection of these loci has provided us with candidates for the underlying functionally active sequence variants for biological experiments to study locus effects and mechanisms of action.

*BCL11A* (B-cell lymphoma/leukaemia 11A) is a known repressor of γ-globin expression. It participates in the haemoglobin switch from γ to β-globin. However, genetic variants in intron 2 of the gene are associated with moderate HbF persistence, detected as increased F-cell numbers and HbF levels. These variants occupy the erythroid enhancer region, characterized by three DNase I-hypersensitive sites (DHSs) situated at +55 kb, +58 kb, and + 62 kb from the transcription start site (TSS) [16].

We detected two haplotypes (defined by rs1427407, rs7565301, and rs7606173) with a strong HbF-increasing effect at this locus. The first (haplotype TGG) carries HbF-increasing alleles at rs1427407 (‘T’) and rs7606173 (‘G’). rs1427407 is located within GATA1 and TAL1 binding motifs at the +62 kb site, suggesting that the T-allele may disrupt GATA1 binding. Thus, rs1427407-‘T’ is the likely causal variant driving the effect of this haplotype. A second haplotype with a significant HbF-increasing effect (haplotype 3) is defined by the presence of the rs7565301 variant (‘A’ allele) and rs7606173 (‘G’). Haplotype 2, carrying only rs7606173 (‘G’) but not rs7565301-‘A’, did not have a significant effect. Hence, we suggest that a causative sequence variant closely linked with rs7565301 likely drives the significant effect of haplotype 3 and possibly a weak (diluted) effect at haplotype 2. We presently cannot exclude that rs7606173 (‘G’) or a closely linked variant caused the HbF-raising effects of haplotypes 1, 2, and 3.

Association of the *BCL11A* locus with HbF has been detected in diverse populations, with different sets of SNPs being reported across studies (Supplementary Table 3) [7, 8, 15, 17]. In our dataset, all these map to the effect haplotypes we have identified. Thus, we suggest that the same underlying causative variants are responsible for all non-anaemic and patient populations studied so far.

The Tanzanian SCD cohort and the African-American cohort of the Cooperative Study of SCD (CSSCD) showed rs1427407 as the strongest association signal with HbF, with rs6545816 and rs7606173 detected as secondary signals [15, 16]. The difference with our findings underscores the influence of population-specific allele frequencies and LD patterns on the identity of lead (sentinel) SNPs reported in specific studies.

At *HBS1L-MYB*, we detected two haplotypes (defined by rs61028892 and rs9399137) with significant HbF-boosting effect. Haplotype 1, tagged by rs9399137-‘C’, causes what has previously been described as the *HMIP-2A* sub-locus [19]. It harbours a 3-bp deletion (rs66650371), which was proposed to be a functional DNA change [20] and was found to disrupt a TAL-1 binding motif within a critical enhancer element for *MYB*, the gene encoding an essential transcriptional regulator of erythropoiesis [21]. Decreased promoter interaction (looping) of an allele carrying the deletion was observed in K562 cells [21], but it is not clear whether this was due to the deletion itself or closely linked variants occupying the same haplotype. HbF-increasing haplotype 2 is defined by the presence of rs61028892-‘C’ and also carries two closely linked alleles, rs148826327-‘A’ (D′ = 1.0, r^2^ = 1.0) and rs116460276-‘G’ (D′ = 1.0, r^2^ = 1.0). It is also linked to a series of weaker variants that previously served to define the *HMIP-2B* sub-locus [19, 25]. rs61028892 is situated at the same *MYB* enhancer element (‘−84 LDB1 site’) as the 3-bp deletion rs66650371 and has previously been proposed as an additional casual variant in the *HMIP* region [26]. This suggests that the HMIP-2 locus might not contain sub-loci, but three functional alleles affecting enhancer element −84, two increasing HbF (rs66650371-del and rs61028892-‘C’) and one representing the baseline, low-HbF state (haplotype ‘**GT**’). The more precise definition of the *HMIP-2* locus we have obtained with these data highlights the significantly lower high-HbF allele frequency at this site in African populations (5.6% total haplotype frequency) compared to individuals of European descent (>20%) and or to patients with substantial European admixture [7, 27].

Consistent with previous studies, HbF variability explained by *BCL11A* and *HMIP* in our study is 24.1%, which is lower than in non-anaemic populations (~ 45%) [7, 12, 13]. Sex and age (up to 3%) are only minor contributors to the overall trait variability. Thus, after major HbF loci are considered, a substantial number of loci with smaller effects or loci that are sickle-cell specific remain to be discovered.

Lastly, we detected three novel HbF loci, *SLC28A3* on chromosome 9, *TICRR*/*KIF7* on chromosome 15, and *PIEZO2* on chromosome 18. *SLC28A3 (solute carrier family 28* member 3) is a sodium-dependent concentrative nucleoside transporter, playing significant roles in physiological processes, including neurotransmission, vascular tone, and transport and metabolism of nucleoside drugs [28, 29]. *TICRR* is a TOPBP1 (Topoisomerase II β-binding protein 1) interacting checkpoint and replication regulator—an essential regulator of DNA replication initiation and S/M and G2/M checkpoints [30]. It is overexpressed in tumour carcinogenesis and progression and was associated with mean corpuscular volume, mean corpuscular haemoglobin, mean corpuscular haemoglobin concentration, body height, and educational attainment in genome-wide association studies [31–33]. *PIEZO2*, on the other hand, is a mechanosensory ion channel (a calcium-permeable mechanosensitive ion channel belonging to the same family as *PIEZO1*) expressed in the primary sensory neurons [34]. It detects mechanical forces and facilitates proprioception, touch perception, lung stretch, and bladder stretch sensation [35, 36]. Mutations in the *PIEZO2* gene have been associated with proprioception defects, respiratory failure, and muscle weakness [37]. The meta-analysed British, Tanzanian, and African-American replication cohorts did not detect these new loci. Possibly, with current sample sizes of sickle cell patient cohorts, loci with marginal evidence for association such as these are difficult to evaluate through pure statistical means. Whether these new loci harbour functional, HbF-affecting variants or reflect spurious trait association will have to be resolved through genetic studies with increased sample size or biological investigation.

In conclusion, we have refined the definition of two major fetal-haemoglobin persistence loci, *BCL11A* and *HS1L-MYB*, which will aid future functional studies that seek to identify new ways to induce HbF expression in patients with sickle cell disease and beta thalassaemia. Finding biological pathways influencing SCD severity remains a major goal for genetic studies with patient populations. The statistical power to detect loci with small effects lies in assembling large patient cohorts and combining worldwide studies.

## Materials and methods

### Study setting and patients

One thousand one hundred and fifty-eight individuals with sickle cell disease (HbSS/HbSβ^0^) recruited from four sites representing three geo-political zones in Nigeria: Lagos University Teaching Hospital, Lagos (South-West); Sickle Cell Foundation Nigeria, Lagos (South-West); University of Abuja Teaching Hospital, Abuja (North-Central); and Ahmadu Bello University Teaching Hospital, Zaria (North-West) (Supplementary Table 1) were genotyped. Patients were excluded from this study if they were on hydroxyurea, younger than five years old, had a blood transfusion in the preceding three months, or had HbSC/HbSβ^+^. A uniform study proforma was completed for data collection across the project sites.

A five-millilitre venous blood sample was collected into an EDTA bottle and processed immediately for complete blood count and haemoglobin profiling. The buffy coat was stored at −20°C for subsequent DNA extraction. Fetal haemoglobin levels were determined using high-performance liquid chromatography (Bio-Rad D-10; Bio-Rad Laboratories, Hercules, CA, USA). The FlexiGene DNA extraction kit (Qiagen GmbH, Hilden, Germany) was used for extraction from buffy coats following the manufacturer’s protocols.

### Genotyping and quality control

Genotyping was performed using the Infinium™ H3Africa Consortium Array containing ~ 2.3 million markers. One thousand one hundred and fifty-eight samples were genotyped and processed in Illumina’s Genome Studio software (version 2.05) for variant calling following the COPILOT raw Illumina genotyping quality control (QC) protocols detailed in [38]. Seventy-seven samples with a genotyping call rate of less than 90% were excluded during the Illumina Genome Studio QC, and no sample was excluded further due to sample quality, as the genotyping call rate was 99.99%. Individual-level QC was carried out to exclude samples with sex discrepancies compared with X-chromosome-derived sex, heterozygosity outliers (heterozygosity ±3 SD from the mean), and genetically identical individuals (identity by descent, pi-hat ~ 1.0) (Supplementary Table 2), retaining 1006 individuals for imputation and downstream analysis. Per-marker QC excluded SNPs with call rate less than 97%, minor allele frequency < 1%, and SNPs that deviated from Hardy–Weinberg equilibrium (*P* < 10^−8^), leaving 1 925 391 autosomal SNPs and X-chromosomes. The overall genotyping rate was 99.99%. Quality control was carried out using PLINK v1.90 (www.cog-genomics.org/plink/1.9/).

To construct the Principal Component Analysis (PCA) of genotypes, we integrated our quality-controlled study dataset with the 1000 Genome reference Phase 3 version 5 [39] after extracting the overlapping markers and excluding the multi-allelic SNPs. The combined data was further filtered (genotype frequency less than 99% and minor allele frequency less than 5%) and pruned (—indep-pairwise 1500 150 0.2) while excluding regions of high linkage disequilibrium before generating the principal components in Plink 2.0 [40]. PCA, inclusive of our dataset, was performed twice: (i) with global populations (CEU: Utah residents with Northern and Western European ancestry for European ancestry, CHB: Han Chinese in Beijing, China, and JPT: Japanese in Tokyo, Japan representing East Asian ancestry, and YRI: Yoruba in Ibadan for African ancestry) and (ii) with a focus on the African continental populations consisting of ESN: Esan in Nigeria; GWD: Gambian in Western Division; LWK: Luhya in Webuye, Kenya; MSL: Mende in Sierra Leone; YRI: Yoruba in Ibadan, Nigeria. Within the continental African PCA plot, our study samples were classified into NG-S (participants enrolled from the South-west Nigeria recruitment site: Lagos) and NG-N (participants enrolled from the North-central (Abuja) and North-west (Zaria) recruitment sites in Nigeria). PCA plots were created in R v.4.2.2.

### Imputation

Filtered genotyped data were used for imputation in Trans-Omics for Precision Medicine (TOPMed) Imputation Server [41] for pre-phasing (with Eagle version 2.4) [42] and imputation (via Minimac4) [43] using the TOPMed panel Version r2 [44]: (https://imputation.biodatacatalyst.nhlbi.nih.gov/#!pages/home).

During post-imputation quality control, SNPs with INFO score < 0.4 and multi-allelic SNPs were excluded, retaining 14 ,816 ,370 SNPs for data analysis.

### Association analysis

To analyse data from a study population that is very diverse in terms of genetic/ethnic background, geographic origin, and age (median age of 15, range 5–60 years), we performed linear-mixed effects regression as implemented by GCTA (Genome-wide Complex Trait Analysis software, version 1.91.7) [45] to control for population stratification by incorporating genetic relationship matrix. This was done using the leave-one chromosome-out approach with age, sex, and recruitment sites as covariates. The natural logarithm of HbF was used to perform association testing. False-discovery rate (FDR) q-values were estimated in R using qvalue [46]; FDR-q values less than 0.05 was considered significant. Manhattan and Q-Q plots were generated in R v.4.2.2 software using the qqman package [47]. *P*-value <  5.0 × 10^−8^ was regarded as being genome-wide significant.

### Conditional analysis, locus association plots, and SNP heritability

Conditional analyses were carried out with conditional and joint analysis in GCTA (GCTA-COJO) using summary statistics [48]. Regional association plots were generated in locus zoom [49]. We computed a stringent genetic score using only the two known major HbF modifier loci present in our population (*BCL11A* and *HBS1L-MYB*) through multiple linear regression modelling in SPSS (version 27), while SNP-heritability was estimated in GCTA [50].

### Replication data set and meta-analysis

To evaluate our putative novel loci and to explore the additional potential gained from assembling a larger dataset through international collaboration, we involved summary-statistical datasets from UK SCD patients studied by us [13] and from HbF GWAS studies in SCD patients performed by researchers in Tanzania [15] and the US [51]. Details of the patients’ recruitment, genotyping/sequencing technologies and platforms, data quality control, imputation, and association analyses for the Tanzanian and African-American datasets have been fully described [15, 51].

The UK SCD GWAS dataset involves 626 HbSS/Sβ^0^ patients and 179 HbSC patients of West African and African Caribbean descent, with sickle mutation genotype included as a covariate during the association analysis. The Tanzanian Muhimbili Sickle Cell Cohort GWAS dataset includes 1187 sickle cell disease (HbSS/Sβ^0^) patients, and the St Jude Children’s Research Hospital (Memphis, TN, US) GWAS dataset includes 584 African-American SCD (HbSS/Sβ^0^) patients. The UKSCD and Tanzania SCD data were analysed using GCTA software with ln [HbF%] as the trait, with correction for age and sex, while the African-American data were analysed with EMMAX software, using square root–transformed HbF% data as the trait [52]. Due to the different transformations of %HbF, sample size weighted meta-analysis (Stouffer’s method in METAL software) [53] with genomic control correction was performed in the SCD patients in the UK, Tanzania, and African-American cohorts. A second meta-analysis, which included the Nigerian cohort, was performed to identify new association signals.

### Fine-mapping

The functional genome-wide complex trait analysis (fGCTA) pipeline runs GCTA-COJO on scaled summary statistics based on the presence or absence of relevant annotations. The annotations used in this analysis are K562 regulatory elements derived using the chromHMM algorithm from Roadmap Epigenome [54, 55], HUDEP-2 obtained from peak calls from Chip-Atlas [56, 57] and GATA1 ChIP-seq on human peripheral derived blood-erythroblast [58]. Using Bayesian methodology, variants that overlap with the annotation have a decreased standard error and vice versa. This pipeline was implemented in C (https://github.com/cgrace1978) and was converted into a prior probability, which is combined with the p-value of the variant to generate a posterior probability.


$$ Posterior=\frac{Prior\ast GWAS\ P}{Total\ Joint} $$

The posterior is then used to generate a new standard error (while keeping the original variant betas). These scaled summary statistics used the GCTA-COJO module that performs a multi-SNP conditional and joint analysis of the summary data [45, 48]. The output of GCTA-COJO contains independent variants in the summary statistics, with a joint p-value below 1 × 10^−5^.

### Haplotype association analysis

Haplotypes were constructed with Haploview 4.2 [59] using phased genotypes from TOPMed Imputation Server. Haplotype effects were estimated using ANOVA in R with adjustment for sex, age, and recruitment sites. Tukey’s post-hoc test was applied for multiple comparisons of means.

Data handling and statistical analyses were performed on the high-performance computing platform CREATE at King’s College London [60].

## Supplementary Material

Supplementary_materials_HMG_revised_ddae014

## Data Availability

The data supporting this study’s findings are available from the corresponding author upon reasonable request.
