# Supplementary material for: The genetic dissection of fetal haemoglobin persistence in sickle cell disease in Nigeria
Source: Hum Mol Genet. 2024 Feb 10;33(10):919–29. doi: 10.1093/hmg/ddae014 (PMC11070134; doi:10.1093/hmg/ddae014)
Supplement: Supplementary_materials_HMG_revised_ddae014 [file supplementary_materials_hmg_revised_ddae014.docx]

**SUPPLEMENTARY TABLES AND FIGURES**


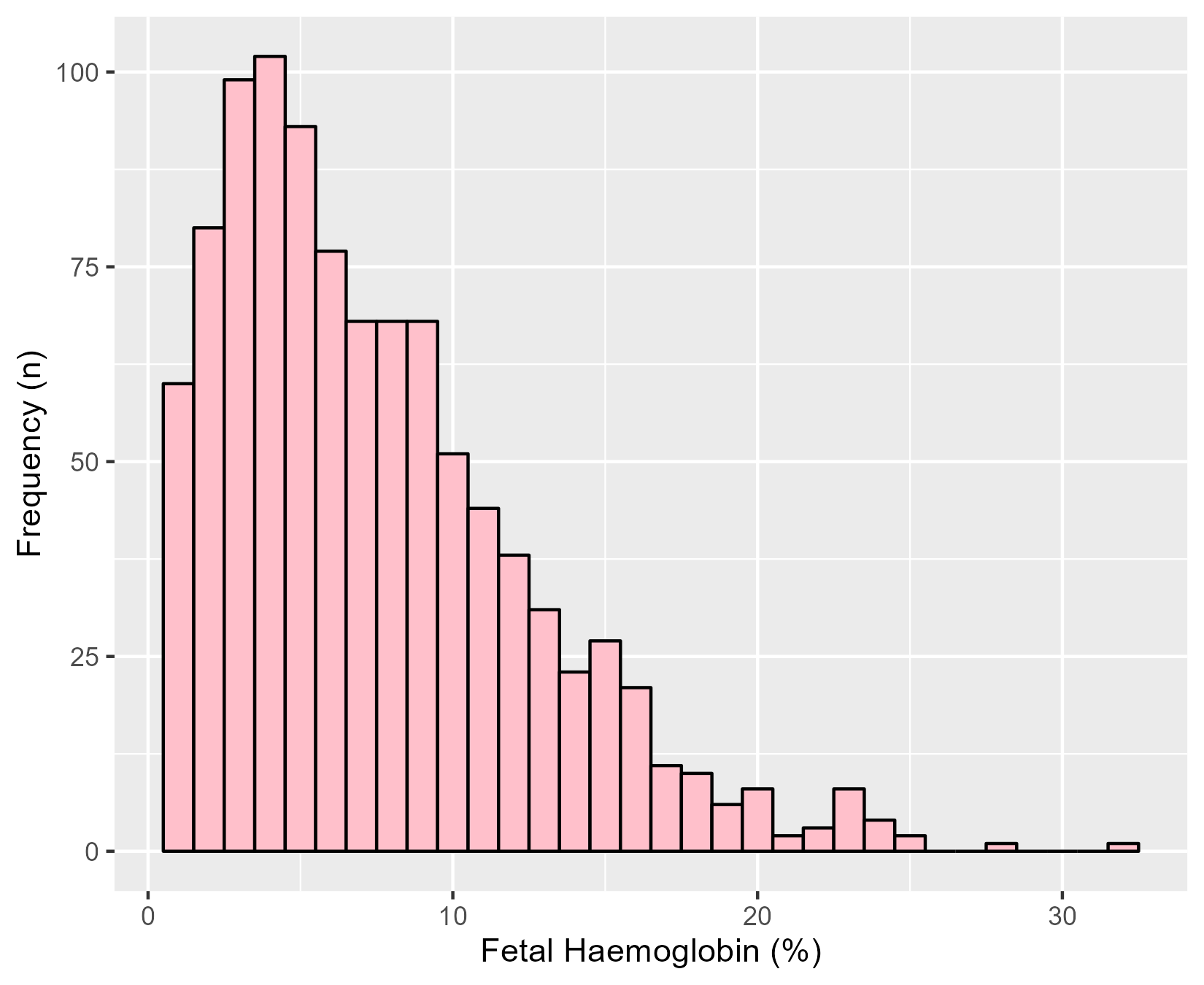


**Supplementary Figure 1. Distribution of Fetal haemoglobin of Nigerian patients with sickle cell disease**

**Supplementary Table 1. Characteristics of the Nigerian cohort included in the GWAS**

|  |  |  | Age (years) | | | HbF (%) | | |
| --- | --- | --- | --- | --- | --- | --- | --- | --- |
|  | **N** | **Sex (M/F)** | **Median [IQR]** | **Min** | **Max** | **Median [IQR]** | **Min** | **Max** |
| ABUJA | 145 | 64/81 | 16[12-22] | 5 | 41 | 9.4 [5.80 – 13.90] | 0.8 | 28.0 |
| LAGOS | 638 | 314/324 | 14 [9 -23] | 5 | 60 | 5.8 [3.40 – 9.30]^a^ | 0.8 | 32.0 |
| ZARIA | 223 | 97/126 | 18 [10-23] | 5 | 45 | 6.1 [4.00 – 10.50]^b^ | 0.8 | 23.3 |
| **TOTAL** | **1006** | **475/531** | **15 [9 -23]** | **5** | **60** | **6.69 [3.67 – 11.02]** | **0.8** | **32.0** |

a: Significantly lower HbF levels compared to patients in ABUJA site (*P* = 1.77 x 10^-9^).

b: Significantly lower HbF levels compared to patients in ABUJA site (*P* = 5.1 x 10^-5^).

The patients were recruited from four sites in Nigeria: two sites in Lagos (South-West), Abuja (North-Central), and Zaria (North-West)


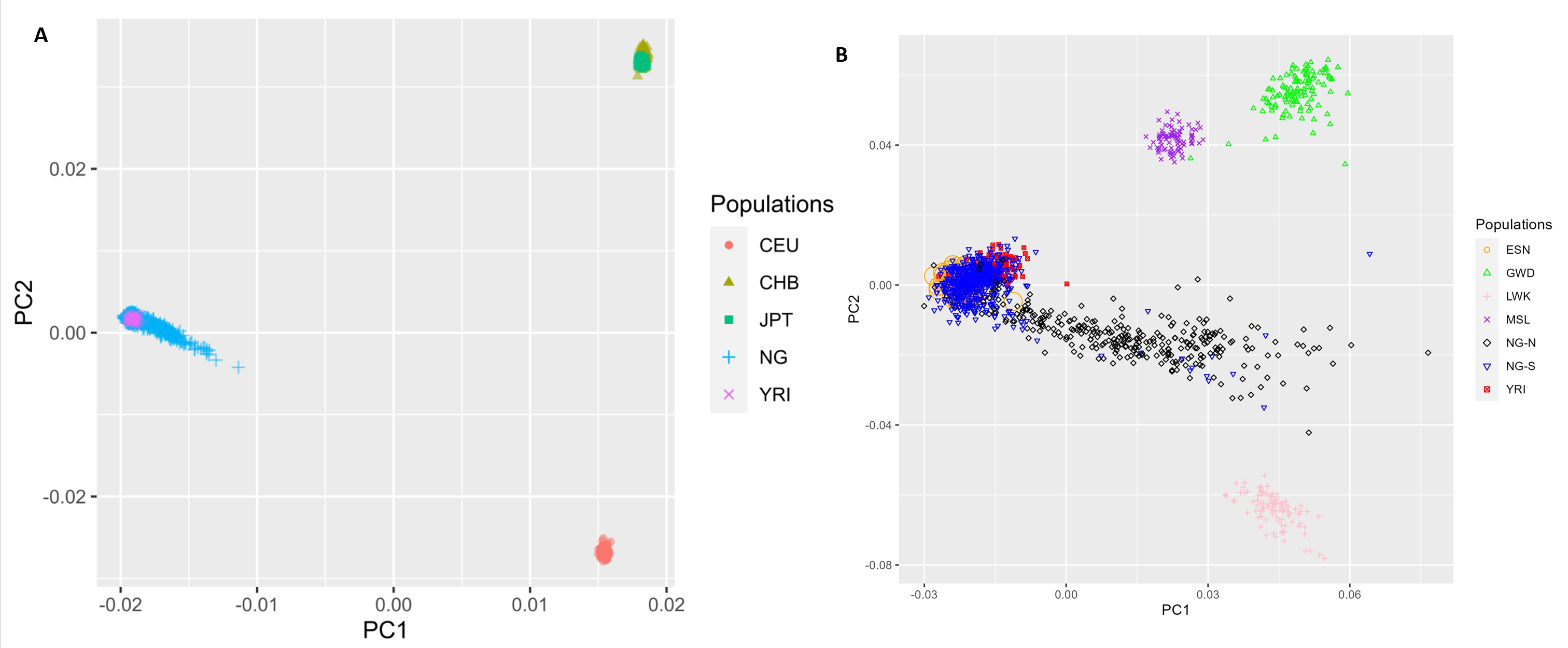


**Supplementary Figure 2. (A) The principal component analysis of study participants with the global populations**. **CEU**: Utah residents with Northern and Western European ancestry representing Europe; **CHB:** Han Chinese in Beijing, China and **JPT**: Japanese in Tokyo, Japan representing East Asia; **NG**: Nigeria (This study); **YRI**: Yoruba in Ibadan representing Africa.

(B)**The principal component analysis of study participants** with the African continental populations. **ESN**: Esan in Nigeria; **GWD**: Gambian in Western Division; **LWK**: Luhya in Webuye, Kenya; **MSL**: Mende in Sierra Leone; **NG-S**: study participants enrolled from the Nigeria South-west recruitment site; **NG-N**: study participants enrolled from the North-central and North-west recruitment sites; **YRI**: Yoruba in Ibadan, Nigeria.

**Supplementary Table 2. Quality control steps**

MAF plot

| **QC step** |  | **Individuals excluded** | **SNPs excluded** |
| --- | --- | --- | --- |
| **Per-individual QC** |  |  |  |
| Genotype call rate < 90%* |  | 77 |  |
| Sex discrepancy samples |  | 21 |  |
| Heterozygosity outliers |  | 20 |  |
| Genetically identical samples |  | 20 |  |
| **SBeta+  **Patients < 5 years  Remaining individuals: **1006** |  | 6  8 |  |
| **Per-marker QC** |  |  |  |
| SNP call rate < 97% |  |  | 61,878 |
| MAF < 1% |  |  | 266,727 |
| HWE p < 10^-8^ |  |  | 5616 SNPs |
| Non-autosomal SNPs |  |  | 44,294 |
| Remaining SNPs: **1,925,391** |  |  |  |

*Samples with genotype call rates < 90% were excluded from the Illumina Genome Studio software following our previously published GWAS quality control protocol. Samples excluded due to heterozygosity outliers were mainly from the region where consanguinity is common. #: Of these, 50941 SNPs were zeroed during pre-QC steps in the Genome studio.

**Supplementary Table 3. Previous HbF-associated genome-wide significant SNPs in three major loci**

| Chr/Gene | Top SNP | MAF | p-value | LD (D’, r^2^) | Sample  size | Population | Cohort | Study type | Authors |
| --- | --- | --- | --- | --- | --- | --- | --- | --- | --- |
| Chr 2:  BCL11A |  |  |  |  |  |  |  |  |  |
|  | rs1427407 | 0.14 | 2.5 x 10^-20^ | 0.94, 0.16 | 179 | Northern Europeans | Twins UK | GWAS | [1] |
|  | rs4671393 | 0.27 | 2.0 x 10^-42^ | 1.0, 0.23 | 1275 | African-American | CSSCD | GWAS | [2] |
|  | rs766432 | 0.28 | 2.61 x 10^-21^ | 1.0, 0.23 | 848 | African-American | CSSCD | GWAS | [3] |
|  | rs766432 | 0.276 | 5.36 x 10^-58^ | 1.0, 0.23 | 2040 | African-American | Multicentre-study | Meta-analysis | [4] |
|  | rs7606173 | 0.45 | 5.14 x 10^-16^ | 1.0, 0.91 | 440 | African_American | SIT Trial | GWAS | [5] |
|  | rs1427407 | 0.22 | 3.74 x 10^-53^ | 0.94, 0.16 | 1213 | East-Africa | Tanzania | GWAS | [6] |
|  | rs1896295 | 0.25 | 2.49 x 10^-26^ | 1.0, 0.22 | 585 | African-American | BCM & SCCRIP | GWAS | [7] |
|  | rs6706648 | 0.41 | 4.96 x 10^-34^ |  | 1006 | West-Africa | Nigeria | GWAS | Current study |
|  |  |  |  |  | |  |  |  |  |

| Chr 6: HBS1L-MYB |  |  |  |  |  |  |  |  |  |
| --- | --- | --- | --- | --- | --- | --- | --- | --- | --- |
|  | rs9399137 | 0.23 | 2.8 x 10^-27^ | 1.0, 0.001 | 179 | Northern Europeans | Twins UK study | GWAS | [1] |
|  | rs9399137 | 0.06 | 5.0 x 10^-11^ | 1.0, 0.001 | 1275 | African-American | CSSCD | GWAS | [2] |
|  | rs9494145 | 0.07 | 4.32 x 10^-17^ | 1.0, 0.59 | 2040 | African-American | Multicentre | Meta-analysis | [4] |
|  | rs9494145 | 0.05 | 2.42 x 10^-10^ | 1.0, 0.59 | 1213 | East-Africa | Tanzania | GWAS | [6] |
|  | rs116460276 | 0.02 | 3.06 x 10^-10^ | 1.0, 1.0 | 585 | African-American | SIT trial | GWAS | [7] |
|  | rs61028892 | 0.02 | 1.18 x 10^-9^ |  | 1006 | West-African | Nigeria | GWAS | Current study |
| Chr 11 |  |  |  |  |  |  |  |  |  |
| *XmnI-HBG2* | rs7482144 | 0.33 | 2.0 x 10^-30^ | - | 179 | Northern Europeans | Twins UK study | GWAS | [1] |
| *XmnI-HBG2* | rs7482144 | 0.07 | 4.0 x 10^-7^ | - | 1275 | African-American | CSSCD | GWAS | [2] |
| OR51B5/OR51B6 | rs5006884 | 0.13 | 4.73 x 10^-8^ | - | 848 | African-American | CSSCD | GWAS | [3] |
|  |  |  |  |  |  |  |  |  |  |

LD (D’, r^2^) relationship between our lead SNP and SNPs reported in previous studies using LDlink: YRI (Yoruba in Ibadan, Nigeria) population in 1000 Genome Project[8]. CSSCD: Cooperative Study of SCD; BCM: Baylor College of Medicine cohort; SCCRIP: St. Jude Children’ Research Hospital Sickle Cell Clinical Research and Intervention Program; SIT: Silent Infarct Transfusion.


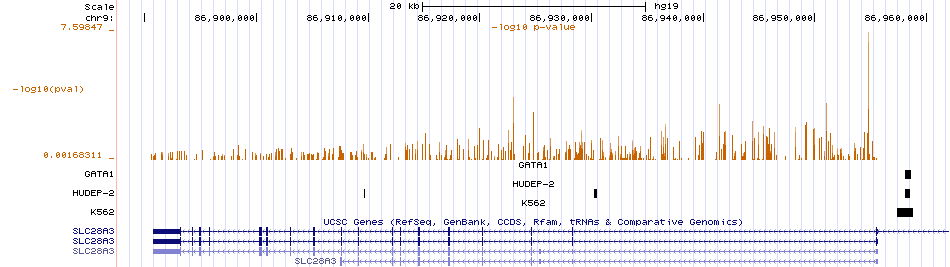


**Supplementary Figure 3:** UCSC plot for *SLC28A3* locus. Tracks in order: log10 p-values for variants; GATA1 ChiP-seq annotation; HUDEP-2 annotation; K562 annotation; genes in this locus.


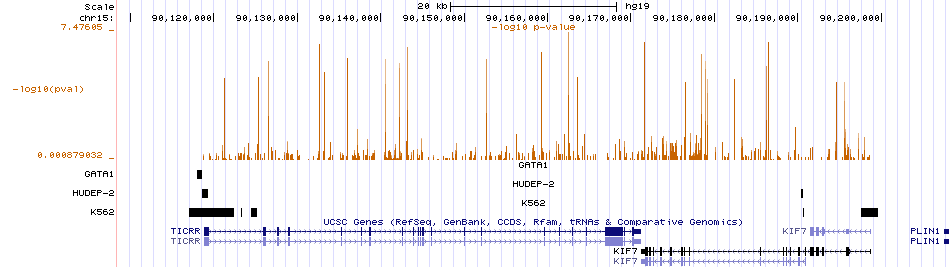


**Supplementary Figure 4:** UCSC plot for *TICRR, KIF7* loci. Tracks in order: log10 p-values for variants; GATA1 ChiP-seq annotation; HUDEP-2 annotation; K562 annotation; genes in this locus.


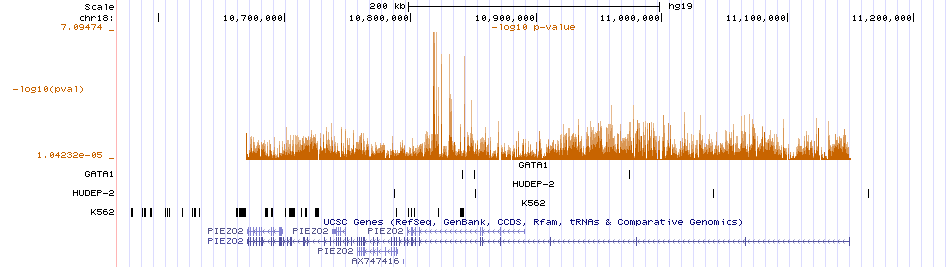


**Supplementary Figure 5:** UCSC plot for *PIEZO2* locus. Tracks in order: log10 p-values for variants; GATA1 ChiP-seq annotation; HUDEP-2 annotation; K562 annotation; genes in this locus.

| **Supplementary Table 4: fGCTA results for the *BCL11A* locus** | | | | | | | | | | | |
| --- | --- | --- | --- | --- | --- | --- | --- | --- | --- | --- | --- |
|  |  |  |  |  | **Scaled Summary Statistics** | | | **COJO output** | | |  |
| **Annotation** | **SNP** | **Chr** | **Position** | **Frequency** | **b** | **se** | **p-value** | **bJ** | **bJ_se** | **pJ** | **Overlap with annotation** |
| GATA1 | rs6706648* | 2 | 60722040 | 0.413 | -0.395 | 0.033 | 1.24 E-33 | -0.288 | 0.039 | 1.32E-13 | FALSE |
| GATA1 | rs1427407 | 2 | 60718043 | 0.253 | 0.409 | 0.037 | 4.57E-28 | 0.257 | 0.044 | 5.88E-09 | TRUE |
| HUDEP-2 | rs6706648* | 2 | 60722040 | 0.413 | -0.395 | 0.033 | 1.24E-33 | -0.292 | 0.039 | 4.98E-14 | FALSE |
| HUDEP-2 | rs1427407 | 2 | 60718043 | 0.253 | 0.409 | 0.038 | 2.86E-27 | 0.254 | 0.045 | 1.21E-08 | FALSE |
| K562 | rs6706648* | 2 | 60722040 | 0.413 | -0.395 | 0.033 | 1.24E-33 | -0.292 | 0.039 | 4.89E-14 | FALSE |
| K562 | rs1427407 | 2 | 60718043 | 0.253 | 0.409 | 0.038 | 2.86E-27 | 0.254 | 0.045 | 1.20E-08 | FALSE |

GCTA-COJO p-value threshold: 1x10^-5^, fGCTA enrichment parameter: x2.5; * Peak SNP for this locus. Position: Chromosomal positions are in hg37;

GATA1: GATA1 ChIP-seq on human peripheral derived blood-erythroblast; b = effect size, SE= standard error, p-value from the scaled summary statistics; bJ = effect size, sbJ_se= standard error and pJ = p-value from a joint analysis of all the selected SNPs.


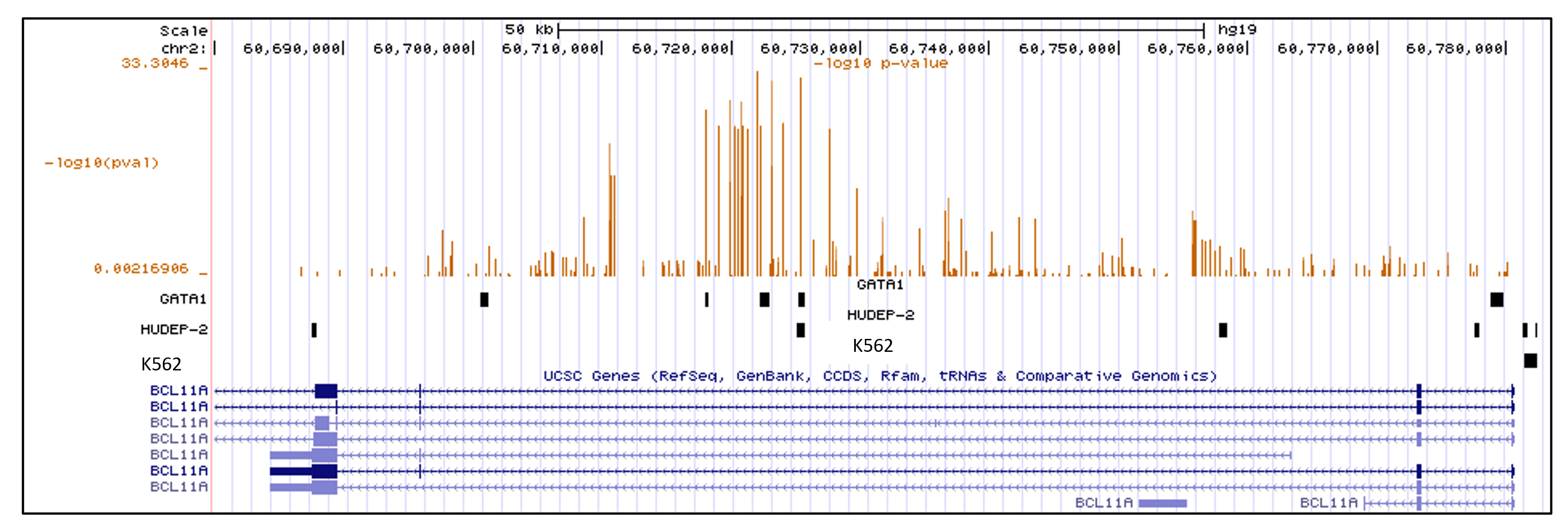


**Supplementary Figure 6:** UCSC plot for the *BCL11A* locus. Tracks in order: log10 p-values for variants; GATA1 ChIP-seq annotation; HUDEP-2 annotation; K562 annotation; genes in this locus.

**Supplementary Table 5. List of SNPs obtained after conditional analysis on *rs1427407*.**

| **Chr** | **rsID** | **bp** | **EA** | **freq** | **β** | **se** | **p** | **βC** | **βC_se** | **pC** |
| --- | --- | --- | --- | --- | --- | --- | --- | --- | --- | --- |
| 2 | rs7565301 | 60496131 | A | 0.30169 | 0.104313 | 0.034342 | 0.002386 | 0.22691 | 0.034482 | 4.69E-11 |
| 2 | rs6706648 | 60494905 | T | 0.412525 | -0.3947 | 0.032454 | 4.96E-34 | -0.23121 | 0.035183 | 4.97E-11 |
| 2 | rs7606173 | 60498316 | C | 0.434292 | -0.39256 | 0.032912 | 8.50E-33 | -0.2344 | 0.035687 | 5.09E-11 |
| 2 | rs6729815 | 60496537 | C | 0.307157 | 0.105949 | 0.034167 | 0.001929 | 0.224669 | 0.034313 | 5.84E-11 |
| 2 | rs7599488 | 60491212 | T | 0.303181 | 0.101711 | 0.034315 | 0.003036 | 0.224745 | 0.034448 | 6.83E-11 |
| 2 | rs6545817 | 60488044 | T | 0.307271 | 0.095021 | 0.034126 | 0.005362 | 0.219086 | 0.03424 | 1.57E-10 |
| 2 | rs6738440 | 60495106 | G | 0.27998 | -0.36236 | 0.035081 | 5.20E-25 | -0.23657 | 0.037066 | 1.75E-10 |
| 2 | rs6709302 | 60500494 | A | 0.313347 | -0.356 | 0.034868 | 1.79E-24 | -0.23542 | 0.036905 | 1.78E-10 |
| 2 | rs10189857 | 60486100 | G | 0.307654 | 0.093908 | 0.034117 | 0.005913 | 0.217889 | 0.034228 | 1.94E-10 |
| 2 | rs13019832 | 60483436 | A | 0.428926 | -0.28073 | 0.031961 | 1.58E-18 | -0.20951 | 0.033245 | 2.93E-10 |
| 2 | rs45606437 | 60495973 | A | 0.341182 | -0.36801 | 0.033736 | 1.05E-27 | -0.22505 | 0.035963 | 3.90E-10 |
| 2 | rs6545816 | 60487726 | C | 0.340457 | 0.079935 | 0.033026 | 0.015506 | 0.207082 | 0.033104 | 3.96E-10 |

Here we showed only p-values ≤ 10^-10^. EA: effect allele; β = effect size; se: standard error; p= pvalue; βC = effect size after conditioning on rs1427407; βC_se: standard error after conditioning on rs1427407; pC = p-value after conditioning on rs1427407.

**Supplementary Table 6. List of selected *BCL11A* SNPs from the 5060 UK Twins**

| Chr | SNP | bp | A1 | A2 | Freq | b | se | p |
| --- | --- | --- | --- | --- | --- | --- | --- | --- |
| 2 | rs10189857 | 60486100 | G | A | 0.426581 | 0.043076 | 0.015578 | 0.00569 |
| 2 | rs6545816 | 60487726 | A | C | 0.456719 | 0.044903 | 0.015558 | 0.003899 |
| 2 | rs6545817 | 60488044 | C | T | 0.458004 | 0.045101 | 0.015567 | 0.003766 |
| 2 | rs1427407 | 60490908 | T | G | 0.152569 | 0.501033 | 0.021644 | 1.49E-118 |
| 2 | rs7599488 | 60491212 | T | C | 0.425 | 0.044047 | 0.01555 | 0.004616 |
| 2 | rs1896294 | 60491939 | C | T | 0.298913 | 0.331639 | 0.016968 | 4.59E-85 |
| 2 | rs766432 | 60492835 | C | A | 0.145455 | 0.505857 | 0.022067 | 2.72E-116 |
| 2 | rs11886868 | 60493111 | C | T | 0.299012 | 0.330788 | 0.016955 | 9.07E-85 |
| 2 | rs4671393 | 60493816 | A | G | 0.144763 | 0.505039 | 0.022111 | 1.80E-115 |
| 2 | rs6706648 | 60494905 | T | C | 0.319071 | -0.24288 | 0.016754 | 1.27E-47 |
| 2 | rs6738440 | 60495106 | G | A | 0.288636 | -0.20583 | 0.017407 | 2.90E-32 |
| 2 | rs7565301 | 60496131 | A | G | 0.266897 | 0.020675 | 0.017494 | 0.237272 |
| 2 | rs6729815 | 60496537 | T | C | 0.463933 | 0.041718 | 0.015478 | 0.007031 |
| 2 | rs1896295 | 60496951 | T | C | 0.144664 | 0.504048 | 0.022052 | 1.25E-115 |
| 2 | rs1896296 | 60496952 | G | T | 0.144565 | 0.503899 | 0.022043 | 1.17E-115 |
| 2 | rs7606173 | 60498316 | C | G | 0.435474 | -0.29618 | 0.015701 | 2.29E-79 |

A1: effect allele; A2: other allele; b = effect size; se: standard error; p = p-value; SNPs in grey refer to rs6706648 and rs7606173 that were investigated for fine-mapping in the white-British Twins’ cohort.

**Supplementary Table 7.** Haplotype association analysis of *BCL11A* SNPs (rs1427407-rs7565301-rs7606173) in the UK SCD patients

| Haplotype | Frequency | HbF effect | P-value |
| --- | --- | --- | --- |
| TGG | 25.3 | 0.561 | < 0.001 |
| GGG | 3.5 | 0.122 | 0.844 |
| GAG | 29.3 | 0.213 | < 0.001 |
| GGC | 40.7 | reference |  |

| **Supplementary Table 8:** fGCTA results for the *HMIP* locus | | | | | | | | | | | |
| --- | --- | --- | --- | --- | --- | --- | --- | --- | --- | --- | --- |
|  |  |  |  |  | **Scaled Summary Statistics** | | | **COJO output** | | |  |
| **Annotation** | **SNP** | **Chr** | **Position_b37** | **Frequency** | **b** | **se** | **p-value** | bJ | bJ_se | pJ | **Overlap with annotation** |
| **GATA1** | rs61028892* | **6** | 135418664 | **0.018** | **0.732** | **0.118** | **4.73E-10** | 0.753 | 0.119 | 2.18E-10 | TRUE |
| **GATA1** | rs66650371 | **6** | 135418632 | **0.032** | **0.514** | **0.087** | **4.12E-09** | 0.531 | 0.088 | 1.74E-09 | TRUE |
| HUDEP-2 | rs61028892* | 6 | 135418664 | 0.018 | 0.732 | 0.118 | 4.73E-10 | 0.753 | 0.119 | 2.14E-10 | TRUE |
| HUDEP-2 | rs66650371 | 6 | 135418632 | 0.032 | 0.514 | 0.087 | 4.12E-09 | 0.531 | 0.088 | 1.72E-09 | TRUE |
| K562 | rs61028892* | 6 | 135418664 | 0.018 | 0.732 | 0.118 | 4.73E-10 | 0.756 | 0.119 | 2.14E-10 | TRUE |
| K562 | rs9399137 | 6 | 135419018 | 0.037 | 0.504 | 0.082 | 6.83E-10 | 0.521 | 0.083 | 3.01E-10 | TRUE |

GCTA-COJO p-value threshold: 1x10^-5^, fGCTA enrichment parameter: x2.5; * Peak SNP for this locus. Position: Chromosomal positions are in hg37;

GATA1: GATA1 ChIP-seq on human peripheral derived blood-erythroblast; b = effect size, SE= standard error, p-value from the scaled summary statistics; bJ = effect size, sbJ_se= standard error and pJ = p-value from a joint analysis of all the selected SNPs.


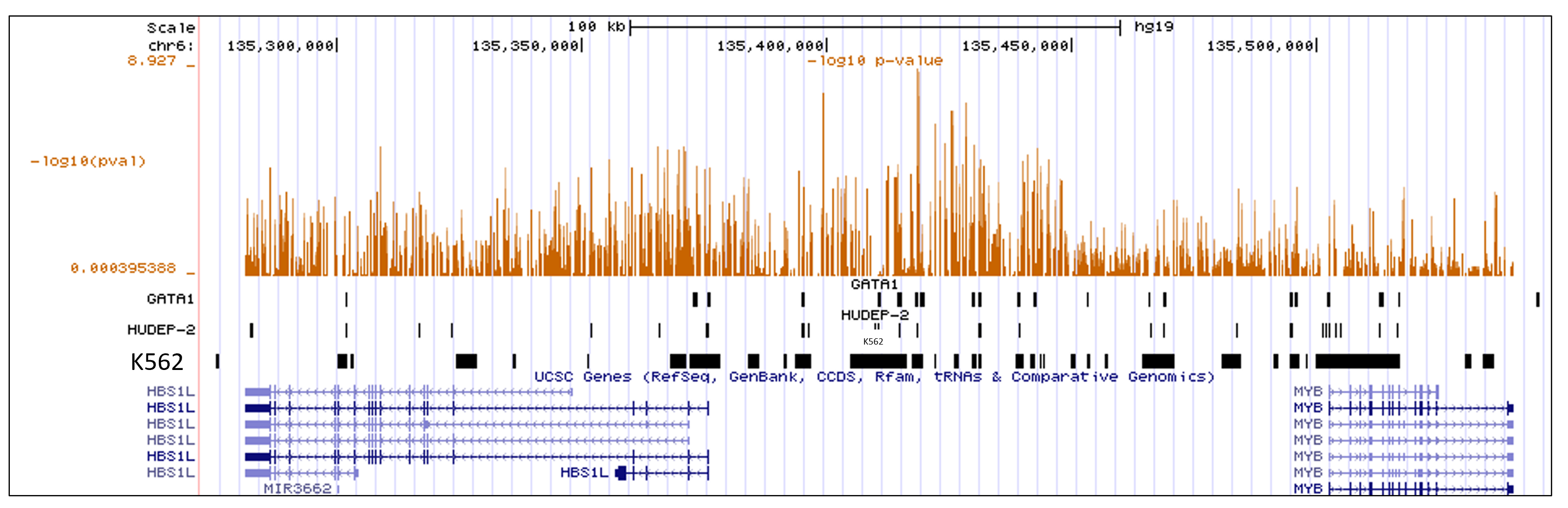


**Supplementary Figure 7:** UCSC plot for the *HMIP* locus. Tracks in order: log10 p-values for variants; GATA1 ChiP-seq annotation; HUDEP-2 annotation; K562 annotation; genes in this locus.

**Supplementary Table 9.** Haplotype association analysis of *HBS1L-MYB* (rs61028892-rs9399137) in the UK SCD patients

| Haplotype | Frequency | HbF effect | P-value |
| --- | --- | --- | --- |
| GC | 5.0 | 0.486 | < 0.001 |
| CT | 1.6 | 0.892 | < 0.001 |
| GT | 93.5 | reference |  |

**Supplementary Table 10. List of all suggestive and genome-wide significant SNPs**

| **chr** | **name** | **bp** | **A1** | **A2** | **freq** | **b** | **se** | **p** |
| --- | --- | --- | --- | --- | --- | --- | --- | --- |
| 2 | rs6706648 | 60494905 | T | C | 0.412525 | -0.3947 | 0.032454 | 4.96E-34 |
| 2 | rs7606173 | 60498316 | C | G | 0.434292 | -0.39256 | 0.032912 | 8.50E-33 |
| 2 | rs45606437 | 60495973 | A | AC | 0.341182 | -0.36801 | 0.033736 | 1.05E-27 |
| 2 | rs1427407 | 60490908 | T | G | 0.252982 | 0.409466 | 0.037563 | 1.14E-27 |
| 2 | rs1896296 | 60496952 | G | T | 0.2715 | 0.38149 | 0.0366 | 1.95E-25 |
| 2 | rs11434093 | 60495961 | C | CA | 0.2695 | 0.383339 | 0.036793 | 2.04E-25 |
| 2 | rs1896295 | 60496951 | T | C | 0.271 | 0.381471 | 0.036691 | 2.56E-25 |
| 2 | rs766432 | 60492835 | C | A | 0.271869 | 0.37791 | 0.036424 | 3.21E-25 |
| 2 | rs1896294 | 60491939 | C | T | 0.271372 | 0.378659 | 0.036553 | 3.80E-25 |
| 2 | rs4671393 | 60493816 | A | G | 0.2735 | 0.377705 | 0.036513 | 4.44E-25 |
| 2 | rs6738440 | 60495106 | G | A | 0.27998 | -0.36236 | 0.035081 | 5.20E-25 |
| 2 | rs11886868 | 60493111 | C | T | 0.269384 | 0.379162 | 0.036752 | 5.92E-25 |
| 2 | rs10195871 | 60493454 | A | G | 0.273857 | 0.373505 | 0.036484 | 1.35E-24 |
| 2 | rs34211119 | 60493183 | GT | G | 0.275 | 0.373377 | 0.036562 | 1.75E-24 |
| 2 | rs10172646 | 60493622 | G | A | 0.275 | 0.373377 | 0.036562 | 1.75E-24 |
| 2 | rs6709302 | 60500494 | A | G | 0.313347 | -0.356 | 0.034868 | 1.79E-24 |
| 2 | rs7557939 | 60494212 | G | A | 0.274775 | 0.373549 | 0.036592 | 1.82E-24 |
| 2 | rs7584113 | 60494176 | A | G | 0.2745 | 0.37325 | 0.036571 | 1.86E-24 |
| 2 | rs13019832 | 60483436 | A | G | 0.428926 | -0.28073 | 0.031961 | 1.58E-18 |
| 2 | rs45484694 | 60483892 | CT | C | 0.230143 | 0.328752 | 0.039205 | 5.05E-17 |
| 2 | rs11692396 | 60483603 | G | A | 0.223161 | 0.327615 | 0.039257 | 7.10E-17 |
| 2 | rs72962585 | 60502567 | G | A | 0.220825 | -0.29849 | 0.038328 | 6.82E-15 |
| 2 | rs58789059 | 60509717 | A | G | 0.172465 | -0.27711 | 0.0417 | 3.03E-11 |
| 2 | rs7340264 | 60509394 | A | G | 0.185572 | -0.26581 | 0.040105 | 3.40E-11 |
| 2 | rs72962596 | 60515110 | T | C | 0.0996 | -0.33283 | 0.052535 | 2.37E-10 |
| 2 | rs72962586 | 60504631 | T | C | 0.101392 | -0.3265 | 0.051566 | 2.42E-10 |
| 2 | rs6732518 | 60481462 | C | T | 0.298387 | 0.226463 | 0.036187 | 3.90E-10 |
| 2 | rs72962592 | 60510666 | T | C | 0.163519 | -0.26563 | 0.042757 | 5.22E-10 |
| 2 | rs72962602 | 60516470 | G | A | 0.164343 | -0.26291 | 0.042616 | 6.86E-10 |
| 2 | rs555276704 | 60528808 | G | GTAA | 0.16633 | -0.26019 | 0.042609 | 1.02E-09 |
| 2 | rs72964419 | 60528663 | T | C | 0.160263 | -0.26403 | 0.043291 | 1.07E-09 |
| 2 | rs79059225 | 60528627 | T | C | 0.164813 | -0.25899 | 0.042683 | 1.30E-09 |
| 2 | rs66488669 | 60507448 | G | A | 0.230343 | -0.21291 | 0.037931 | 1.99E-08 |
| 2 | rs4672393 | 60470519 | A | C | 0.379602 | -0.18147 | 0.032941 | 3.61E-08 |
| 2 | rs114125602 | 60513084 | A | G | 0.027638 | -0.5223 | 0.096339 | 5.91E-08 |
| 2 | rs72964414 | 60523168 | T | C | 0.148851 | -0.21822 | 0.044088 | 7.44E-07 |
| 6 | rs61028892 | 135097526 | C | G | 0.017982 | 0.732037 | 0.120351 | 1.18E-09 |
| 6 | rs9399137 | 135097880 | C | T | 0.037276 | 0.503912 | 0.083658 | 1.71E-09 |
| 6 | rs35786788 | 135097904 | A | G | 0.037276 | 0.503912 | 0.083658 | 1.71E-09 |
| 6 | rs66650371 | 135097494 | T | TTAC | 0.032096 | 0.513867 | 0.089748 | 1.03E-08 |
| 6 | rs148826327 | 135078218 | A | G | 0.016 | 0.723182 | 0.127556 | 1.43E-08 |
| 6 | rs115099895 | 135104618 | A | G | 0.034328 | 0.464427 | 0.086831 | 8.86E-08 |
| 6 | rs114603312 | 135104880 | C | T | 0.034328 | 0.464427 | 0.086831 | 8.86E-08 |
| 6 | rs114398597 | 135107536 | G | A | 0.034363 | 0.463423 | 0.086834 | 9.45E-08 |
| 9 | rs115555854 | 84339933 | A | C | 0.014955 | -0.72726 | 0.130523 | 2.52E-08 |
| 11 | rs1406381 | 62301213 | G | T | 0.320344 | -0.16572 | 0.03341 | 7.04E-07 |
| 14 | rs191465524 | 52721225 | G | T | 0.015936 | -0.63712 | 0.123409 | 2.44E-07 |
| 14 | rs188152509 | 52740738 | G | C | 0.01592 | -0.63708 | 0.123406 | 2.44E-07 |
| 15 | rs140496989 | 89619296 | A | G | 0.045817 | -0.42712 | 0.077342 | 3.34E-08 |
| 15 | rs78432130 | 89643261 | G | T | 0.045862 | -0.40726 | 0.077303 | 1.38E-07 |
| 15 | rs137993810 | 89628452 | G | C | 0.043327 | -0.41851 | 0.079446 | 1.38E-07 |
| 15 | rs78973372 | 89589437 | T | G | 0.045364 | -0.40542 | 0.077696 | 1.81E-07 |
| 15 | rs146039045 | 89616003 | A | G | 0.055224 | -0.3489 | 0.069713 | 5.59E-07 |
| 16 | rs188481235 | 1446101 | T | C | 0.019901 | -0.54714 | 0.111648 | 9.56E-07 |
| 18 | rs58817161 | 10818373 | C | T | 0.017396 | -0.63287 | 0.117935 | 8.04E-08 |
| 18 | rs142386135 | 10819008 | G | A | 0.017396 | -0.63287 | 0.117935 | 8.04E-08 |
| 18 | rs143311786 | 10820712 | G | A | 0.017396 | -0.63287 | 0.117935 | 8.04E-08 |


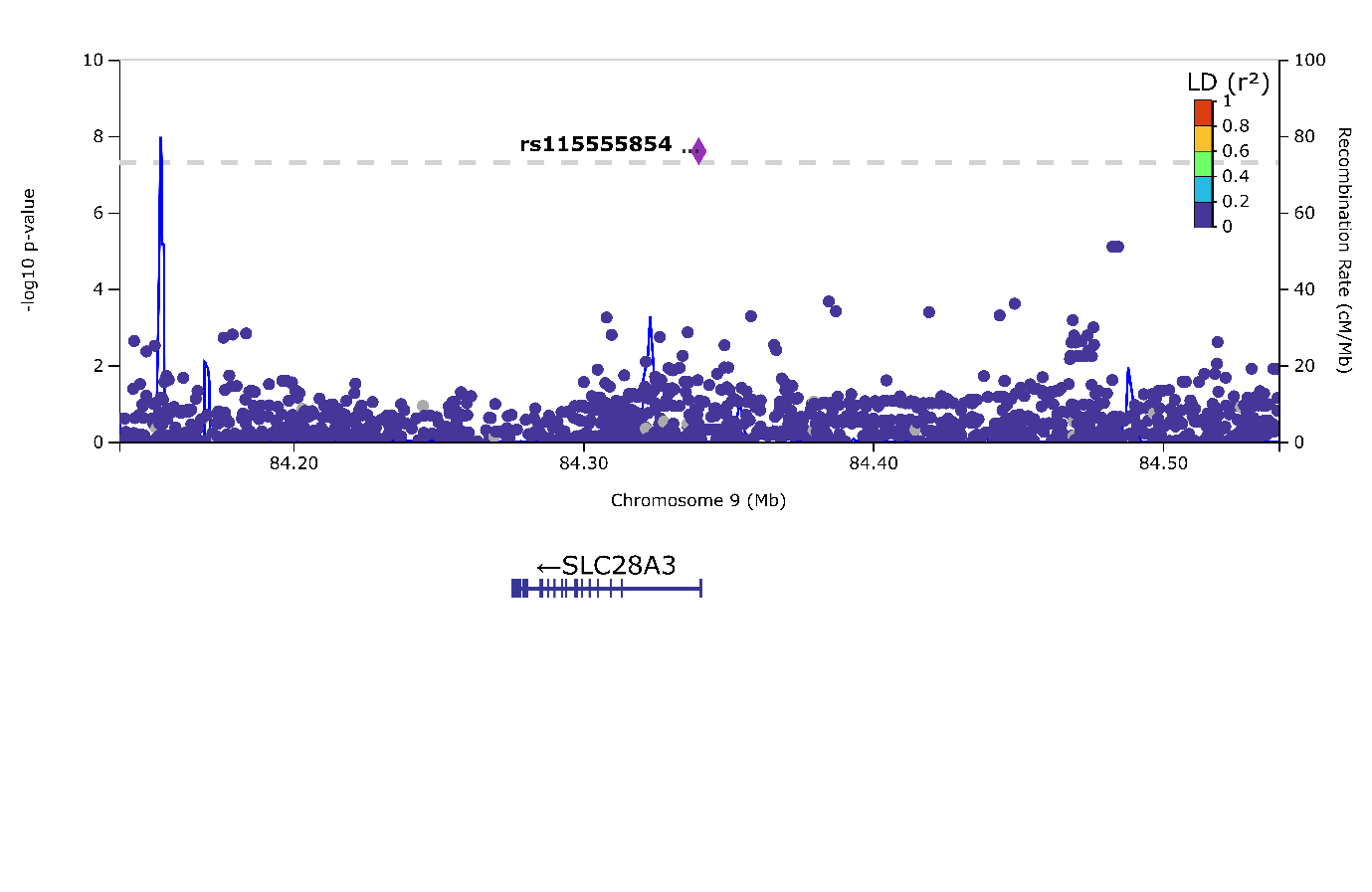


**Supplementary Figure 8.** Regional Association plot of *SLC28A3*


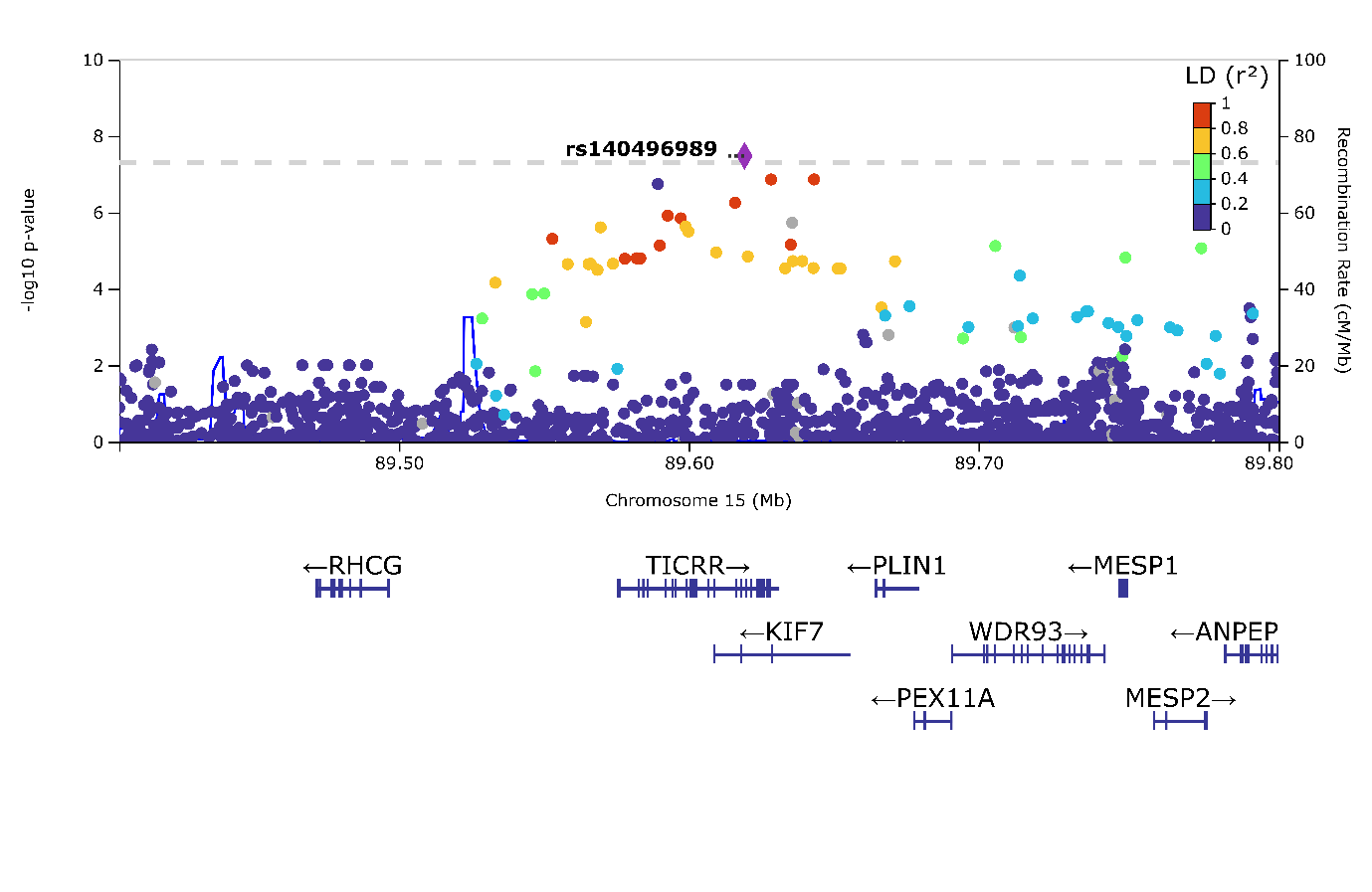


**Supplementary Figure 9.** Regional Association plot of *TICRR/KIF7*


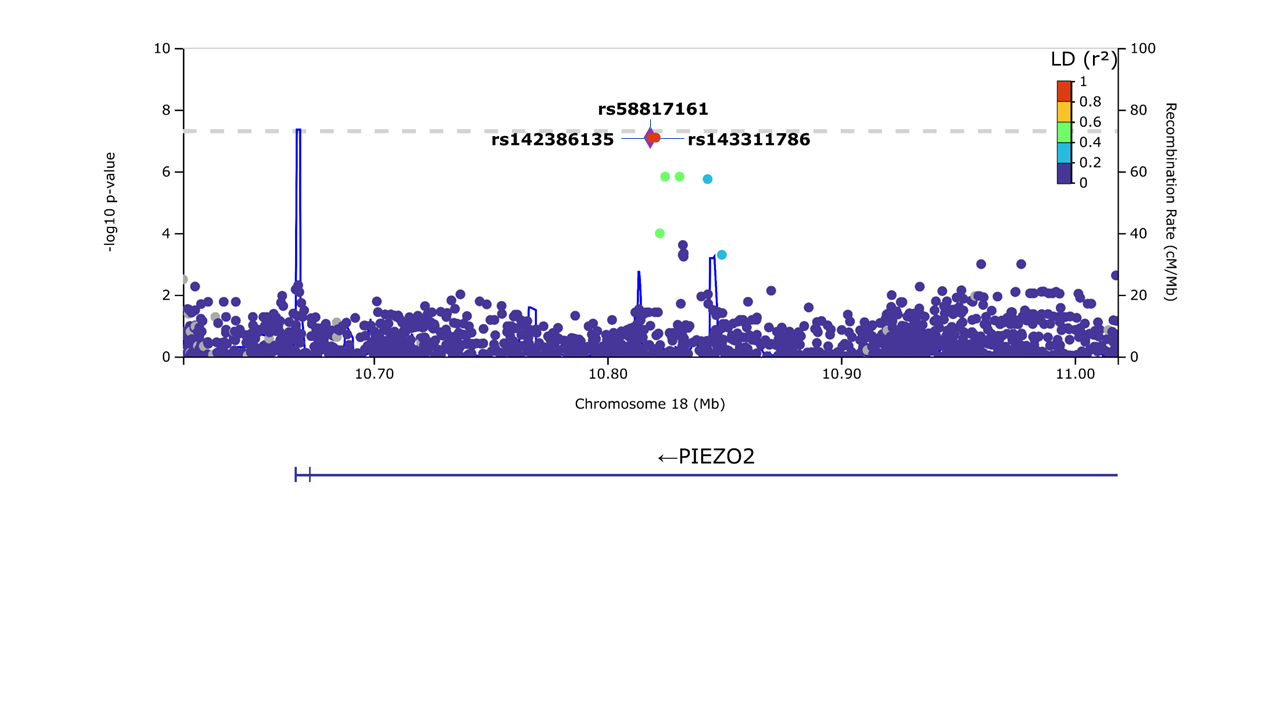


**Supplementary Figure 10.** Regional Association plot of *PIEZO2*

**Supplementary Table 11. Summary statistics of African-American data for replication**

|  |  |  |  |  | **African-American cohort** | | | | | | |
| --- | --- | --- | --- | --- | --- | --- | --- | --- | --- | --- | --- |
|  | CCDC154 |  |  |  | **Cross-sectional (BCM & SCCRIP)** | | | **Longitudinal^1^ (N = 326)** | | **Longitudinal, conditioned on score^2^ (N = 326)** | |
| **Chr** | **Nearest gene** | **SNP** | **Position** | **EA/OA** | **EAF** | **β** | **P-value** | **β** | **P-value** | **β** | **P-value** |
| 9 | *SLC28A3* | rs115555854 | 84339933 | A/C | 0.01 | **-** | - | -0.53 | 0.16 | -0.38 | 0.25 |
| 11 | *SCGB1D4* | rs1406381 | 62301213 | G/T | 0.45 | -0.05 | 0.341 | 0.043 | 0.57 | 0.025 | 0.72 |
| 14 | *PSMC6* | rs191465524 | 52721225 | G/T | 0.01 | -0.17 | 0.467 | -0.2 | 0.64 | -0.21 | 0.58 |
| 14 | *STYX* | rs188152509 | 52740738 | G/C | 0.01 | -0.17 | 0.467 | -0.2 | 0.64 | -0.21 | 0.58 |
| 15 | *TICRR* | rs78973372 | 89589437 | T/G | 0.02 | 0.04 | 0.836 | 0.27 | 0.19 | 0.23 | 0.22 |
| 15 | *TICRR, KIF7* | rs146039045 | 89616003 | A/G | 0.03 | -0.03 | 0.826 | 0.27 | 0.17 | 0.25 | 0.16 |
| 15 | *TICRR, KIF7* | rs140496989 | 89619296 | A/G | 0.02 | 0.04 | 0.836 | 0.25 | 0.17 | 0.23 | 0.18 |
| 15 | *TICRR, KIF7* | rs137993810 | 89628452 | G/C | 0.02 | 0.05 | 0.773 | 0.31 | 0.14 | 0.27 | 0.16 |
| 15 | *KIF7* | rs78432130 | 89643261 | G/T | 0.02 | 0.04 | 0.836 | 0.27 | 0.19 | 0.23 | 0.22 |
| 16 | *CLCN7, CCDC154* | rs188481235 | 1446101 | T/C | 0.02 | 0.15 | 0.485 | 0.54 | 0.06 | 0.38 | 0.15 |
| 18 | *PIEZO2* | rs58817161 | 10818373 | C/T | 0.02 | -0.06 | 0.774 | -2.4 | 0.00071 | -2.5 | 0.00028 |
| 18 | *PIEZO2* | rs142386135 | 10819008 | G/A | 0.01 | -0.15 | 0.534 | -2.4 | 0.00071 | -2.5 | 0.00028 |
| 18 | *PIEZO2* | rs143311786 | 10820712 | G/A | 0.02 | -0.06 | 0.774 | -0.24 | 0.00071 | -2.5 | 0.00028 |

Chr: Chromosome; SNP: reference ID for the Single Nucleotide Polymorphism; EA: Effect allele; OA: Other allele; EAF: Effect allele frequency; β: allelic effect size; N = sample size; BCM: Baylor College of Medicine cohort; SCCRIP: St. Jude Children’ Research Hospital Sickle Cell Clinical Research and Intervention Program.

1: Longitudinal analysis of HbF at ages 1-6: (6 measurements per person, for a total of 1956 data points).  The analysis was adjusted for age, sex, Hydroxyurea therapy, principal components 1-5, and the SNP-age interaction; 2: Longitudinal analysis, adjusting for age, sex, Hydroxyurea therapy, principal components 1-5, the SNP-age interaction, and 11-SNP polygenic score for HbF (PGS^HbF^) as detailed in [7].

**Supplementary Table 12. Genome-wide significant and suggestive loci obtained from the meta-analysis.**

| **Chr** | **Locus** | **SNP** | **Position** | **EA/OA** | **EAF** | **N** | **p-value** | **Direction of effect allele** |
| --- | --- | --- | --- | --- | --- | --- | --- | --- |
| 1 | *WDR78* | rs77479749 | 66828256 | C/T | 0.089 | 3554 | 5.74 x 10^-7^ | + |
| 2 | *BCL11A* | rs1427407 | 60490908 | T/G | 0.237 | 3572 | 1.83 x 10^-109^ | + |
| 2 | *TEX51* | rs116591810 | 126849099 | T/C | 0.035 | 3546 | 5.54 x 10^-7^ | - |
| 4 | *GRID2* | rs539530558 | 93555532 | T/G | 0.01 | 1759 | 4.67 x 10^-7^ | - |
| 5 | *AC01460.1* | rs1560177 | 23322878 | C/T | 0.189 | 3562 | 6.5 x 10^-7^ | + |
| 6 | *HBS1L-MYB* | rs9399137 | 135097880 | C/T | 0.044 | 3562 | 1.77 x 10^-27^ | + |
| 6 | *NKAIN2* | rs138298800 | 124625064 | A/G | 0.011 | 1179 | 2.69 x 10^-7^ | - |
| 6 | *ATP5MGP2* | rs149569206 | 122875198 | A/T | 0.016 | 3504 | 1.16 x 10^-7^ | - |
| 9 | *SLC28A3* | rs115555854 | 84339933 | A/C | 0.015 | 1003 | 2.73 x 10^-8^ | - |
| 11 | *OR52S1P* | rs4531463 | 5078160 | A/G | 0.074 | 1375 | 2.03 x 10^-10^ | + |
| 14 | *AC005520.4* | rs115521884 | 73844597 | C/A | 0.079 | 355 | 9.25 x 10^-7^ | + |
| 17 | *FAAP100* | rs12675 | 81540246 | A/G | 0.272 | 2926 | 7.69 x 10^-7^ | + |
| 21 | *AF254983.2* | rs79114373 | 10399098 | T/C | 0.117 | 579 | 8.25 x 10^-7^ | - |

Chr: Chromosome; SNP: reference ID for the Single Nucleotide Polymorphism; EA: Effect allele; OA: Other allele; EAF: Effect allele frequency; N = sample size.

**References**

1. Menzel S, Garner C, Gut I, Matsuda F, Yamaguchi M, Heath S, Foglio M, Zelenika D, Boland A, Rooks H, et al: **A QTL influencing F cell production maps to a gene encoding a zinc-finger protein on chromosome 2p15.** *Nat. Genet.* 2007, **39:**1197-1199.

2. Lettre G, Sankaran VG, Bezerra MA, Araujo AS, Uda M, Sanna S, Cao A, Schlessinger D, Costa FF, Hirschhorn JN, Orkin SH: **DNA polymorphisms at the BCL11A, HBS1L-MYB, and beta-globin loci associate with fetal hemoglobin levels and pain crises in sickle cell disease.** *Proc. Natl. Acad. Sci. U S A* 2008, **105:**11869-11874.

3. Solovieff N, Milton JN, Hartley SW, Sherva R, Sebastiani P, Dworkis DA, Klings ES, Farrer LA, Garrett ME, Ashley-Koch A, et al: **Fetal hemoglobin in sickle cell anemia: genome-wide association studies suggest a regulatory region in the 5' olfactory receptor gene cluster.** *Blood* 2010, **115:**1815-1822.

4. Bae HT, Baldwin CT, Sebastiani P, Telen MJ, Ashley-Koch A, Garrett M, Hooper WC, Bean CJ, Debaun MR, Arking DE, et al: **Meta-analysis of 2040 sickle cell anemia patients: BCL11A and HBS1L-MYB are the major modifiers of HbF in African Americans.** *Blood* 2012, **120:**1961-1962.

5. Bhatnagar P, Purvis S, Barron-Casella E, DeBaun MR, Casella JF, Arking DE, Keefer JR: **Genome-wide association study identifies genetic variants influencing F-cell levels in sickle-cell patients.** *Journal of Human Genetics* 2011, **56:**316-323.

6. Mtatiro SN, Singh T, Rooks H, Mgaya J, Mariki H, Soka D, Mmbando B, Msaki E, Kolder I, Thein SL, et al: **Genome wide association study of fetal hemoglobin in sickle cell anemia in Tanzania.** *PLoS One* 2014, **9:**e111464.

7. Rampersaud E, Kang G, Palmer LE, Rashkin SR, Wang S, Bi W, Alberts NM, Anghelescu D, Barton M, Birch K, et al: **A polygenic score for acute vaso-occlusive pain in pediatric sickle cell disease.** *Blood Adv.* 2021, **5:**2839-2851.

8. Machiela MJ, Chanock SJ: **LDlink: a web-based application for exploring population-specific haplotype structure and linking correlated alleles of possible functional variants.** *Bioinformatics* 2015, **31:**3555-3557.
